# JAK/STAT inhibition reprograms T cell activation and metabolism in inflammatory arthritis patients

**DOI:** 10.1007/s00011-026-02242-5

**Published:** 2026-05-05

**Authors:** Viviana Marzaioli, Aenea A. I. Brugman, Niamh O’Dowd, Achilleas Floudas, Aine Gorman, Carl Orr, Douglas J. Veale, Ursula Fearon

**Affiliations:** 1 Molecular Rheumatology, School of Medicine, Trinity Biomedical Sciences Institute, TCD, Dublin, Ireland; 2 EULAR Centre of Excellence, Centre for Arthritis and Rheumatic Diseases, St Vincent’s University Hospital, UCD, Dublin, Ireland

**Keywords:** Inflammatory arthritis, JAK/STAT inhibition, T cells, Polyfunctionality, Metabolism

## Abstract

**Objectives:**

Inflammatory arthritis (IA) is a group of autoimmune diseases characterised by joint inflammation and progressive damage, thus impairing the patient’s quality of life. The JAK/STAT pathway inhibitor Tofacitinib has been successfully introduced into the clinic to treat patients with IA, however its direct effect on T cell responses is widely unknown. This study aims to assess the effect of Tofacitinib on T cell activation, polyfunctionality, proliferation and metabolism.

**Methods:**

The effect of Tofacitinib on T cells from peripheral blood, synovial fluid and synovial tissue was evaluated with multidimensional flow cytometric analysis. T cell proliferation was assessed by flow cytometry and T cell metabolism was examined by qPCR and Seahorse XF analyser. To investigate the effect of Tofacitinib on T cell polarisation, naïve T cells were differentiated into Th1, Th2 and Th17 with specific cytokine cocktails. Soluble mediators were evaluated by MSD multiplex analysis.

**Results:**

Tofacitinib significantly inhibited T helper cell activation as evidenced by a marked reduction in the frequency of PD-1/CD69/CD25-positive cells (*p* < 0.01). Reduced activation was consistent with impairment of pathogenic polyfunctionality of peripheral blood and synovial tissue-derived T cells. The impact of Tofacitinib on T cell plasticity was further substantiated by reduced T cell polarisation towards Th1 (*p* < 0.05), Th2 (*p* < 0.05), Th17 (*p* < 0.05) and a reduction in genes associated with T cell functions. The attenuation of pathogenic T cell responses is linked to metabolic adaptation, with Tofacitinib leading to a switch in metabolic capacity, mainly ascribed to the CD4^−^CD8^+^ T cell compartment.

**Conclusions:**

Tofacitinib strongly alters T cell responses and potentially limits T cell pathogenicity by decreasing their activation, polyfunctionality, differentiation, and metabolic potential in both the circulation and the joints of patients with inflammatory arthritis.

**Supplementary Information:**

The online version contains supplementary material available at 10.1007/s00011-026-02242-5.

## Introduction

Inflammatory arthritis (IA), including Rheumatoid (RA) and Psoriatic arthritis (PsA), are chronic systemic inflammatory diseases that cause progressive damage to the joint, leading to functional disabilities and a significant decrease in the patient’s quality of life [1–3]. While significant progress has been made in treatment strategies for patients living with IA over the last two decades, including the development of therapies targeting anti-tumour necrosis factor (TNF)α, cluster of differentiation (CD)20, interleukin (IL)-6R, IL-17, and IL-12/IL-23, there is still a significant proportion of patients who have suboptimal responses or experience intolerance to current therapies [4–6]. The newest class of drugs target the Janus kinase (JAK)/signal transducer and activator of transcription (STAT) pathway, which has been shown to have a key role in driving numerous pathogenic mechanisms implicated in IA disease. JAK/STAT inhibitors are small molecules, offering the option of long-term oral biologic treatment for both RA and PsA [7, 8]. However, a better understanding of the immunoregulatory effect of these molecules is required. We and others have shown increased expression of STAT signalling components in RA and PsA synovial biopsies, synovial fibroblasts (FLS) and T cells [9, 10]. Furthermore, Tofacitinib, a JAK1/3 and selective JAK2 inhibitor, has been demonstrated to inhibit spontaneous release of pro-inflammatory cytokines from ex vivo RA/PsA synovial explant cultures, in addition to inhibition of FLS migratory and invasive capacity [9, 11–13]. Interestingly, the effect of Tofacitinib on FLS pathogenic function is associated with a shift in their metabolic profile [11, 14] and with reciprocal interactions between key transcriptional signalling pathways, including pSTAT-3, hypoxia inducible factor (HIF)-1α, and glycolytic enzymes in cancer and inflammatory cells [14, 15].

Studies examining the effect of JAKi on the regulation of innate immune cell development have shown that Tofacitinib impairs in vitro monocyte-derived dendritic cell (DC) development and activation through the regulation of NADPH oxidase 5 (NOX5) activity [16], and ex vivo on CD209/CD14^+^ DC, thus reducing the infiltration of pathogenic DC into the joint [17]. In addition, Tofacitinib has been shown to decrease the T cell stimulatory capability of DC [17, 18]. A phase II/III randomised clinical trial in patients with RA has shown that Tofacitinib leads to the suppression of CD4^+^CD8^−^ T cell proliferation in the periphery [19], and more recently, it has been shown that Tofacitinib may increase T cell senescence in RA [20]. However, the effect of JAK/STAT inhibition on T cell activation in IA remains largely unexplored.

Dysregulated T cell responses characterised by the emergence of polyfunctional T cells are a hallmark of synovial inflammation [21–23]. Indeed, T cell polyfunctionality profiles have been observed in endotypes of RA [24]. In addition, a positive correlation between disease activity and synovial T cell polyfunctionality has been demonstrated in RA and PsA, and similar profiles have been observed prior to disease onset in individuals at risk of developing RA [22–26].

This study aims to investigate the effect of Tofacitinib on immune cell function, with a specific focus on key facets of T cell responses, including activation, proliferation, differentiation, polyfunctionality, and metabolism, both in the periphery and importantly, at the site of inflammation in patients with IA. We demonstrate that Tofacitinib impacts T cell pathogenicity on multiple levels, leading to decreased activation, polyfunctionality, and differentiation, while inducing a reversion of the glycolytic switch, especially in CD4^−^CD8^+^ T cells.

## Materials and methods

### Patient recruitment and sample collection

Blood samples were obtained from patients with active IA through the Rheumatology Department at St. Vincent’s University Hospital, Dublin, Ireland, and collected in lithium heparin tubes (BD). Patients who met the 2010 American College of Rheumatology (ACR)/European Alliance of Associations for Rheumatology (EULAR) for RA and Classification Criteria for PsA (CASPAR) were included in this study. If they did not fill the specific classification criteria for RA or PsA, they were classified as unclassified inflammatory arthritis (uIA). Synovial fluid (SF) and tissue (ST) biopsies were obtained by key-hole video arthroscopic surgery at the rheumatology clinics of St. Vincent’s University Hospital, Dublin, Ireland. Ethical approval for this study was obtained by the St. Vincent's University Hospital and Medical Research Committees, and the study was performed in accordance with the Declaration of Helsinki. All patients gave fully informed written consent. The total number of patients enrolled in this study was n = 37, comprising unclassified inflammatory arthritis (uIA, 24.32%), PsA (35.14%), and RA (40.54%). Patient demographics can be found in Table 1.

**Table 1:**
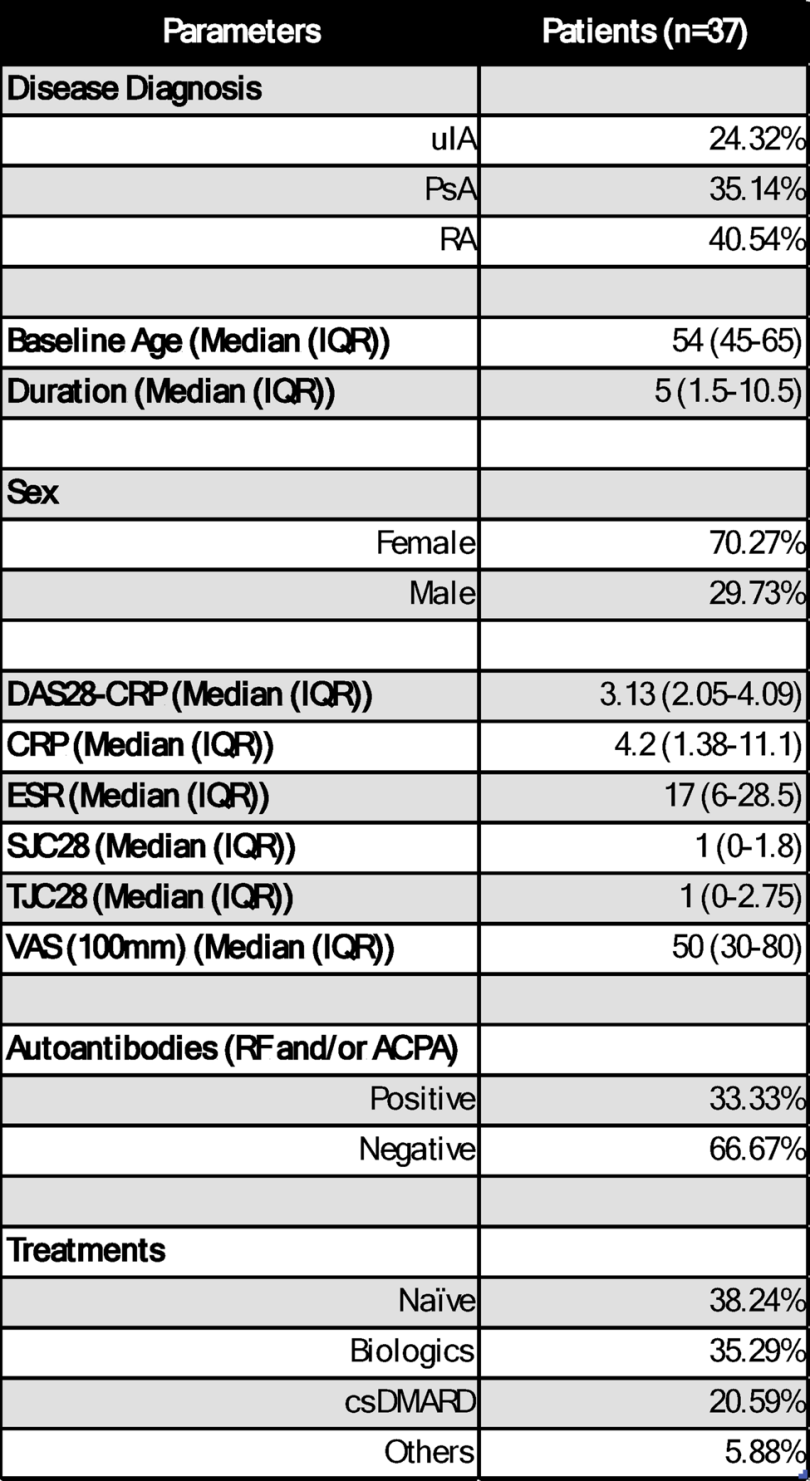
Patients’ demographics. A total of n = 37 IA patients were enrolled in this study. Patients’ diagnosis, sex, disease active, disease duration, seropositivity and treatment are displayed. Two patients were on Tofacitinib treatment at the time of blood collection but still displayed active disease status

### Peripheral blood and synovial fluid mononuclear cell isolation and synovial tissue digestion

Peripheral blood mononuclear cells (PBMC) and synovial fluid mononuclear cells (SFMC) were isolated by density gradient centrifugation (Lymphoprep, Stemcell Technologies) according to the manufacturer’s recommendations. ST biopsies obtained at the time of arthroscopy were mechanically and enzymatically digested using the GentleMacs dissociator and a soft tumour dissociation kit (Miltenyi Biotec, Germany), as previously described [23, 25].

### Flow cytometric analysis

Cells from PBMC, SFMC, and ST cell suspensions were plated at 3 × 10^5^ cells/well in a flat bottom 96-well plate in 100μL of RPMI 1640 (cRPMI; Sigma), supplemented with 10% foetal bovine serum (FBS), HEPES (20 mM), Pencillin/Streptomycin (100 unit/mL and 100 μg/mL), and Amphotericin B (0.25 μg/mL) (Gibco-BRL, UK). Cells were treated with 5 μM Tofacitinib (or dimethyl sulfoxide (DMSO) as control) or 1 µg/mL Humira (Adalimumab, anti-TNFα) (or immunoglobulin (Ig)G as control) in the presence of anti-CD3 (1 μg/mL) and anti-CD28 (2 μg/mL) (Thermofisher) for three days. Subsequently, cells were stimulated with cell stimulation cocktail (phorbol 12-myristate 13-acetate (PMA)/Ionomycin) (eBiosciences) for 1 h prior to the addition of Brefeldin-A (BFA) and Monensin as per the manufacturer’s instructions (ThermoFisher) for a further 4 h incubation.

Single-cell suspensions were collected and washed in ice-cold phosphate-buffered saline (PBS) prior to incubation with LIVE/DEAD™ Fixable Near-IR dye (BD Biosciences). An Fc receptor-blocking step was performed by incubating the cells with TruStain FcX™ Fc blocking solution (BioLegend). Cells were then stained for 30 min at 4 °C with the surface marker antibodies (Table 2). For intracellular staining, the eBioscience™ Forkhead box P3 (Foxp3)/Transcription Factor Staining Buffer Set (Lifesciences) was used as per the manufacturer’s protocol. Cell viability in response to Tofacitinib was similar to DMSO control (Supplementary Fig. 1A).

**Table 2:**
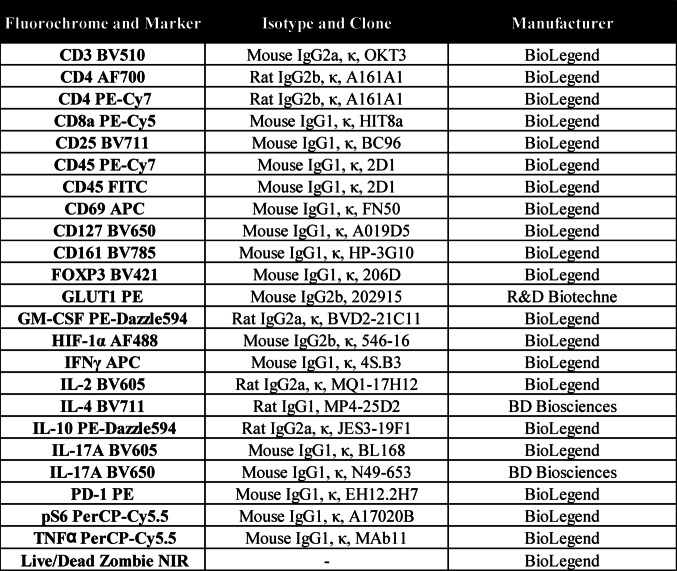
Antibody panel utilised for flow cytometric analysis

Samples were acquired using the Fortessa LSR II or the Aurora Flow Cytometer (BD) and analysed using Flowjo software v10.7 (Treestar Inc.). T cell proliferation was assessed 5 days post initial activation using the CellTrace™ Violet (CTV) assay and FlowJo v10 cell proliferation plugin to calculate the proliferation index.

### Simplified presentation of incredibly complex evaluations analysis

To examine co-expression of markers, the supervised algorithm Simplified Presentation of Incredibly Complex Evaluations (SPICE, Version 5.1) was used. SPICE is a software which enables the analysis of large data sets from polychromatic flow cytometry and organises the data graphically. A Boolean gating strategy was adopted to identify all possible cell populations and imported into the SPICE programme. Pie charts generated as a result of this analysis represent frequency of marker co-expression, where each pie segment represents a specific marker combination, as per relevant legend. Each arc represents a single marker (colour-coded as per legends), single costimulatory marker-positive cells are represented by individual arcs surrounding each pie chart, whereas marker co-expression is illustrated by overlapping arcs on top of the relative pie segment [16, 17, 26].

### U-plex multiplex assay

Isolated PBMC and ST cell suspensions were treated with 5 μM Tofacitinib (or DMSO as control) in the presence of anti-CD3 (1 μg/mL) and anti-CD28 (2 μg/mL) for three days. Supernatants were harvested and cytokines (interferon (IFN)γ, IL-21, IL-22, IL-4, IL-9, TNFα) were analysed by Meso Scale discovery (MSD) multiplex assay (Meso Scale Diagnostics, USA) according to the manufacturer’s protocol.

### Naïve T cell polarisation

T cells from patients with IA were enriched using an untouched T cell isolation kit (Miltenyi Biotec) according to the manufacturer’s instructions. Following enrichment, naïve CD4^+^ T cells were obtained using one of two approaches. In experiments requiring high-purity naïve populations, enriched T cells were stained with extracellular antibodies against CD3, CD4, CD8, CD45RO, and C–C chemokine receptor (CCR)-7 and fluorescence-activated cell sorting (FACS) was performed to isolate CD3^+^CD4^+^CD45RO⁻CCR7^+^ naïve T cells. Alternatively, naïve CD4^+^ T cells were isolated using a naïve CD4^+^ T cell magnetic isolation kit (Miltenyi Biotec), according to the manufacturer’s instructions. Following isolation, naïve CD4^+^ T cells were cultured in 200 µL of cRPMI per well at a density of 1 × 10^6^ cells/mL in round-bottom 96-well plates (Greiner Bio-One) in the presence of 5 × 10^5^ cells/mL of lethally irradiated allogeneic PBMC (St James’s Hospital, Transfusion Department). Naïve T cells were stimulated with anti-CD3/anti-CD28/anti-CD2 activation beads (Treg Suppression Inspector, Miltenyi Biotec) according to the manufacturer’s instructions. For T helper (Th)1 and Th2 differentiation conditions, human CellXVivo™ Th1 and Th2 differentiation kits were used, respectively, according to the manufacturer’s instructions (R&D Systems). For Th17-polarising conditions, naïve T cells were cultured with IL-1β (20 ng/mL), IL-6 (30 ng/mL), IL-23 (30 ng/mL), transforming growth factor (TGF)-β1 (2.5 ng/mL), and anti-IFNγ and anti-IL-4 blocking antibodies (1 µg/mL and 2.5 µg/mL, respectively) (all from Miltenyi Biotec). Naïve CD4^+^ T cells were cultured for 14 days with 50% media changes performed on days 3, 6, 9, and 11. To assess the effect of Tofacitinib on T cell polarisation, cultures were supplemented with 5 µM Tofacitinib (or DMSO as control) on days 0 and 6. Evaluation of Th polarisation was performed by flow cytometric analysis and intracellular cytokine staining following stimulation with PMA/ionomycin in the presence of BFA/monensin, as described above.

### Pan T cell isolation

Negative selection of T cells was performed using the Pan T Cell Isolation Kit (Miltenyi Biotec, Germany). Freshly isolated PBMC were resuspended in MACS buffer and incubated with a biotin-antibody cocktail targeting non-T cells (CD14, CD15, CD16, CD19, CD34, CD36, CD56, CD123, and CD235a) for 5 min at 4 °C. This was followed by incubation with anti-biotin and anti-CD61-conjugated magnetic microbeads for 10 min at 4 °C. The labelled non-T cells were retained on an LS column in a MACS separator, while the unlabelled pan T cells were collected in the flow-through. Pan T cells were plated at a density of 3 × 10^5^ cells/well in cRPMI in a 48-well plate and treated with 5 μM Tofacitinib (or DMSO as control) for 24 h and used for downstream applications.

### Seahorse XF metabolic analysis

A Mitochondrial Stress Test was performed using the Seahorse XFe96 Analyser (Agilent) to assess the metabolic profile of T cells in response to Tofacitinib. Isolated pan T cells were seeded at 3 × 10^5^ cells/well in a 96-well Seahorse microplate (Agilent Technologies), left to rest for 1 h before 5 μM Tofacitinib (or DMSO control) stimulation for a further 24 h. Subsequently, cells were incubated with Seahorse DMEM (Agilent), supplemented with 1.8 g/L glucose (Biosciences), 1 mM sodium pyruvate (Biosciences) and 0.8 M L-glutamine (Biosciences) for 1 h at 37 °C in non-CO_2_ humidified air. Basal oxidative phosphorylation (OxPhos) and glycolysis levels were measured by five baseline oxygen consumption rate (OCR) and extracellular acidification rate (ECAR) readings, respectively, before oligomycin (2 μg/mL; Tocris), trifluorocarbonylcyanide phenylhydrazone (FCCP; mitochondrial uncoupler; 5 μM; Sigma-Aldrich), antimycin A (complex III inhibitor; 2 μM; Abcam), and rotenone (2 μM; Sigma-Aldrich) were injected to assess the metabolic capacity of pan T cells. Three OCR and ECAR readings were taken per inhibitor injection.

### Quantitative RT-PCR

Total RNA was extracted from pan T cells (isolated as above) using a miRNeasy Kit (Qiagen, Valencia, CA, USA). For mRNA analysis, cDNA was synthesised with a high‐capacity cDNA reverse transcription kit (Applied Biosystems). Quantitative reverse transcription-polymerase chain reaction (qRT-PCR) was performed with SYBR green qPCR master mix (Applied Biosystems, Foster City, CA, USA) with the specific primers listed in Table 3. The relative level of each target transcript was calculated using the 2^–ΔΔCt^ method, with RPLP0 as the housekeeping gene.

**Table 3:** Primer sequences used in this study

Gene Name	Forward primer	Reverse primer
BTLA	5’-CAGATGTAAAAAGTGCCTCAGAAC	5’- TAAACGATACAGGAGCCAGGG
CCR7	5’-ACC TGG GTA TGC CTG TGT CAA	5’-AGA CTC GAA CAA AGT GTA GTC CA
COX7B	5’-AGCCACCAGAAACGTACACC	5’-TAACTCTGCCAACAGGGGAC
CXCR4	5’- CACGCCACCAACAGTCAGAG	5’-AGTCGGGAATAGTCAGCAGGA
GLUT1	5’-CTTCCAGTATGTGGAGCAACTGT	5’-GCACAGTGAAGATGATGAAGACG
GZMB	5’- TGCAGGAAGATCGAAAGTGCG	5’-GAGGCATGCCATTGTTTCGTC
HIF-1α	5’-GAAACTTCTGGATGCTGGTGATTT	5’-GCAATTCATCTGTGCTTTCATGTCA
HK2	5’-TTCTTGTCTCAGATTGAGAGTGAC	5’-TTGCAGGATGGCTCGGACTTG
NCAM1	5’-TGTCCGATTCATAGTCCTGTCC	5’-CTCACAGCGATAAGTGCCCTC
RPLP0	5’-GCGTCCTCGTGGAAGTGACATCG	5’-TCAGGGATTGCCACGCAGGG
SUCNR	5’-TCTCAACTTGGTCATCATGGC	5’-GCGTGAAGCGATCCTCACATT
UQCRB	5’-GGATGTTTCGCATTAAGAGGGC	5’-TGGTCCACTGCTCTTTAGGC

### Statistical analysis

Statistical analysis was performed using Prism version 10 (GraphPad Software). Data are represented as median with interquartile range (IQR) or connecting lines. Non-parametric paired two-tailed t test (Wilcoxon) were used as indicated. One-way and two-way analysis of variance (ANOVA) with multiple comparisons were applied when comparing more than two groups. Statistical significance was considered with p values of less than 0.05.

## Results

### Tofacitinib inhibits T cell activation in the circulation of patients with IA

**Fig. 1:**
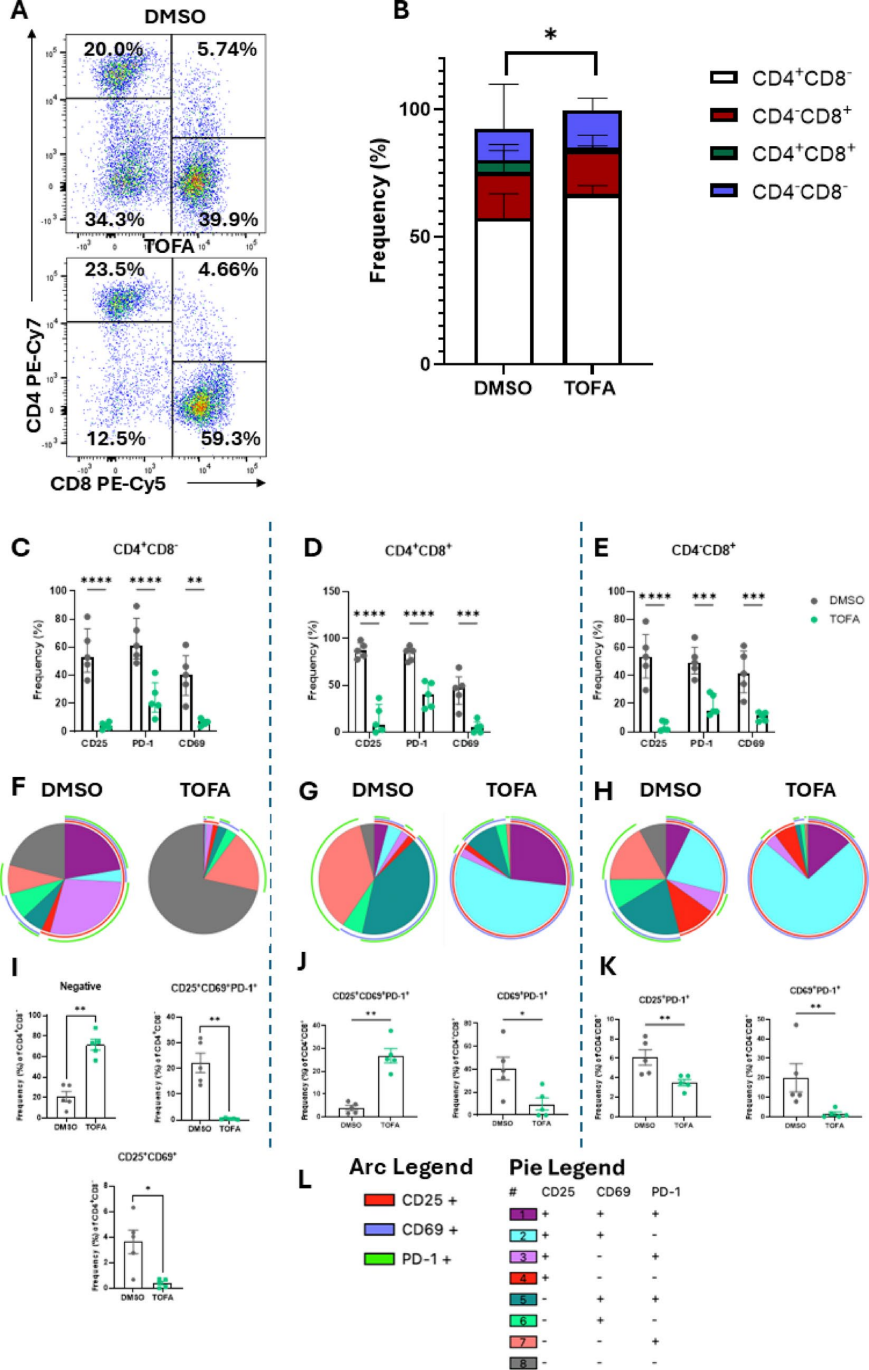
Tofacitinib inhibits circulatory T cell activation in patients with IA. PBMC were isolated from IA patients and treated with 5 µM Tofacitinib (or DMSO as control) in the presence of anti-CD3/anti-CD28 for 3 days. **A** Representative T cell population frequency dot plot and **B** Stacked bar chart (median with IQR) for CD4^+^CD8^−^, CD4^+^CD8^+^, CD4^−^CD8^+^ and CD4^−^CD8^−^ population frequencies (corresponding graphs in Supplementary Fig. 2A) (n = 10; RA n = 5, PsA n = 4, uIA n = 1). Frequency of CD25, CD69 and PD-1 bar graphs (median with IQR) in **C** (n = 5; RA n = 2, PsA n = 1, uIA n = 1) CD4^+^CD8^−^, **D** CD4^+^CD8^+^, and **E** CD4^−^CD8^+^. SPICE analysis for CD25, CD69 and PD-1 in **F** CD4^+^CD8^−^, **G** CD4^+^CD8^+^, and **H** CD4^−^CD8^+^ populations; overlapping arcs represent co-expression of markers. Co-expression of different combinations of activation markers is expressed as median with IQR in **I** CD4^+^CD8^−^, **J** CD4^+^CD8^+^, and **K** CD4^−^CD8^+^ populations. **L** Legend for arc and pie. Differences among groups were evaluated by One-way or Two-way ANOVA with multiple comparisons (Bonferroni correction). For two group comparisons, paired Wilcoxon test was used. *p < 0.05. **p < 0.01, ***p < 0.001, ****p < 0.0001

To investigate the effect of Tofacitinib on T cell activation in vitro, PBMC were isolated from patients with IA and treated with 5 µM Tofacitinib (or DMSO as control) in the presence of anti-CD3/anti-CD28 for 3 days. Cells were then collected and subjected to flow cytometric analysis for specific T cell activation markers (CD25, CD69, programmed cell death protein (PD)-1). T cells subsets were identified as CD45^+^CD3^+^, using CD4 and CD8 antibodies for subset identification (gating strategy in Supplementary Fig. 1B). We firstly investigated whether JAK/STAT inhibition altered the frequencies of the T cell subsets in circulation (Fig. 1A and B). Interestingly, Tofacitinib led to a significant redistribution of CD4/CD8 T cell subsets, with a significant increase in the CD4^+^CD8^−^ population (Fig. 1A and B and Supplementary Fig. 2A *p* < 0.05), mirrored by a significant decrease in the CD4^+^CD8^+^ population (Fig. 1A and B and Supplementary Fig. 2A *p* < 0.01), which correlated with disease activity score DAS28-CRP (*p* < 0.01, Supplementary Table 1). This effect was unique to JAK/STAT inhibition, as no difference in T cell distribution was observed in T cells treated with Humira (TNFα inhibitor, Supplementary Fig. 3A).

Different studies have shown that activated T cells are enriched in the joints of patients with RA, with PD-1- and CD69-expressing cells correlating with disease progression [27, 28], while CD4^+^CD25^bright^ T cell have been reported to accumulate in inflamed joints in chronic rheumatic disease [29]. Here, we show that Tofacitinib significantly decreased the frequency of CD25 (*p* < 0.0001), PD-1 (*p* < 0.0001) and CD69 (*p* < 0.01) expressing CD4^+^CD8^−^ T cells (Fig. 1C and Supplementary Fig. 2C). A similar behaviour was observed in CD4^+^CD8^+^ (Fig. 1D,* p* < 0.0001 for CD25 and PD-1, and *p* < 0.001 for CD69) and CD4^−^CD8^+^ (Fig. 1E,* p* < 0.0001 for CD25, *p* < 0.001 for CD69 and PD-1). Interestingly, the decrease in CD25^+^ T cells did not result in a significant decrease of the regulatory T cell (Treg) population (gated as CD4^+^CD127^−^CD25^+^FoxP3^+^, as observed in Supplementary Fig. 4A and B), indicating that the reduction in CD25^+^ cells likely reflect changes in activated T cells rather than Tregs. This suggests that Tofacitinib has a strong effect in inhibiting aberrant T cell activation, an effect that is unique to JAK/STAT inhibition, as no effect is observed in the presence of TNFα inhibitors (Supplementary Fig. 3B).

To evaluate co-expression of activation markers on T cell phenotypes, we performed SPICE analysis, where the pie segments represent the frequency of T cells expressing different combinations of markers, and the arcs represent the expression of CD25, CD69, or PD-1 activation markers; overlapping arcs indicate simultaneous marker expression (Fig. 1F–K, legends in Fig. 1L). In CD4^+^CD8^−^ T cells, Tofacitinib led to a significant increase (*p* < 0.01) in the frequency of T cells not expressing any activation markers (negative fraction Fig. 1F and I), paralleled by a decrease in different combinations of markers co-expressed together. In particular, we observed a significant decrease in co-expression of all three markers together CD25^+^CD69^+^PD-1^+^ (*p* < 0.01) and of CD25^+^CD69^+^ (*p* < 0.05). Interestingly, in CD4^+^CD8^+^ T cells, although the single expressed markers were all decreased, we observed an increase in the co-expression of all markers CD25^+^CD69^+^PD-1^+^ in the presence of Tofacitinib (Fig. 1G and J, *p* < 0.01), which could be a compensatory mechanism. However, in this subset we observed a decrease in the CD69^+^PD-1^+^ population (*p* < 0.05). In CD4^−^CD8^+^ cells, a significant decrease was observed in the CD25^+^PD-1^+^ (*p* < 0.01) and the CD69^+^PD-1^+^ populations (*p* < 0.01) (Fig. 1H and K). Altogether, this suggests that Tofacitinib decreases the activation state of circulatory T cell subsets in patients with IA, with distinct effects observed across the three T cell subpopulations.

### Tofacitinib inhibits the polyfunctionality of IA-patient circulating T cells

**Fig. 2:**
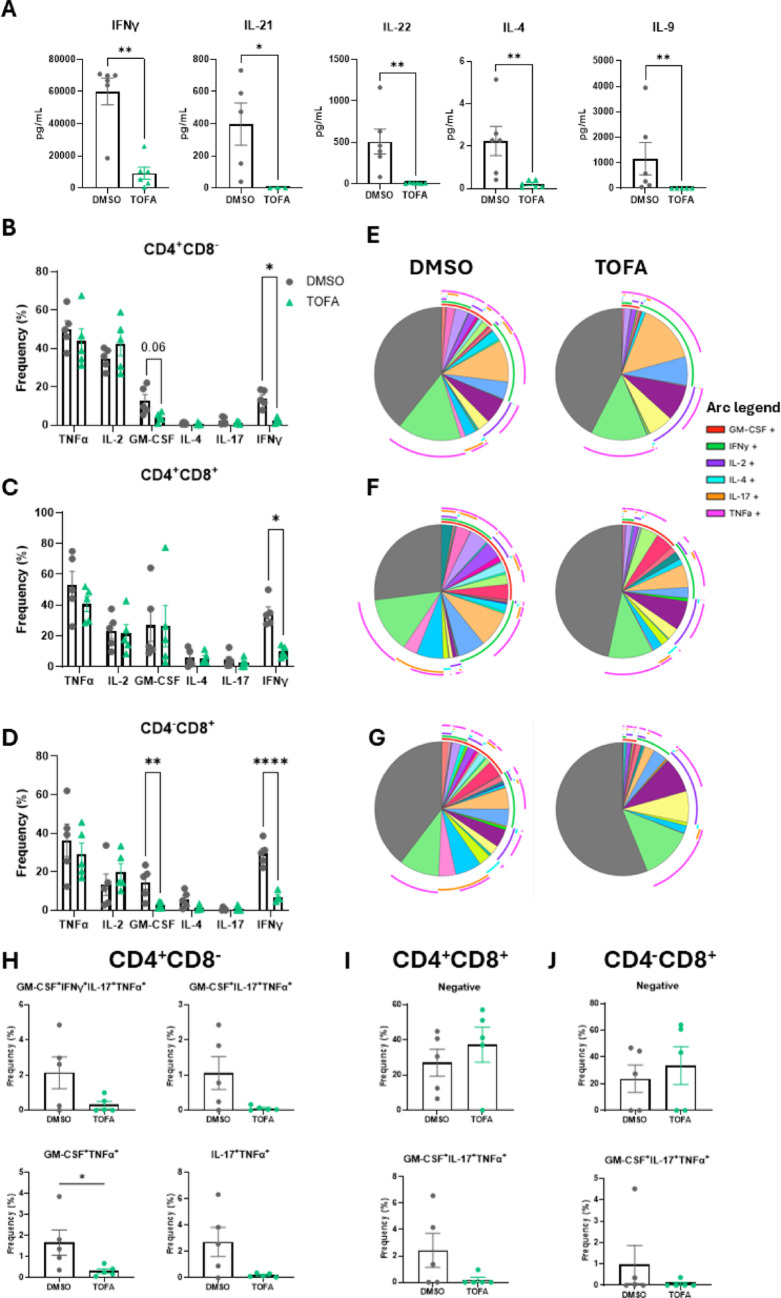
Tofacitinib decreases T cell polyfunctionality in the circulation of IA patients. PBMC were isolated from IA patients (n = 5; RA n = 4, PsA n = 1) and treated with 5 µM Tofacitinib (or DMSO as control) in the presence of anti-CD3/anti-CD28 for 3 days. **A** Supernatants were collected, and multiplex analysis was performed for IFN-γ, IL-21, IL-22, IL-4, and IL-9. **B**–**J** Cytokine release was stimulated with PMA/Ionomycin for 1 h, followed by BFA/Monensin for further 4 h. Surface and intracellular staining were performed for GM-CSF, IFN-γ, IL-2, IL-4, IL-17A, and TNFα. Bar charts are represented as median with IQR for **B** for CD4^+^CD8^−^, **C** CD4^+^CD8^+^, and **D** CD4^−^CD8^+^ populations. SPICE analysis in **E** CD4^+^CD8^−^, **F** CD4^+^CD8^+^, and **G)** CD4^−^CD8^+^ populations are shown, with overlapping arcs representing co-expression of markers (legend for pie chart in Supplementary Fig. 5B). Co-expression of different combinations of activation markers is expressed as median with IQR in **H** CD4^+^CD8^−^, **I** CD4^+^CD8^+^, and **J** CD4^−^CD8^+^ populations. Differences among groups were evaluated by One-way or Two-way ANOVA with multiple comparisons (Bonferroni correction). For two group comparisons, paired Wilcoxon test was used. *p < 0.05. **p < 0.01, ***p < 0.001, ****p < 0.0001

Previous studies have identified a strong correlation between T cell polyfunctionality in RA and PsA with disease activity and progression [22, 23, 26, 30], therefore, we next investigated the effect of Tofacitinib on T cell cytokine release, expression, and polyfunctionality in patients with IA. PBMC from patients with IA were treated with 5 µM Tofacitinib or DMSO in the presence of anti-CD3/anti-CD28 for 3 days. Soluble proteins were measured in the supernatants using a multiplex assay. Interestingly, Tofacitinib led to a significant decrease in T cell secretion of multiple soluble cytokines (Fig. 2A), including IFNγ, IL-22, IL-4, IL-9 (all *p* < 0.01) and IL-21 (*p* < 0.05). The cells were then further stimulated with PMA/Ionomycin as detailed in the methods, to measure intracellular cytokine production by flow cytometric analysis. We observed a significant decrease in CD4^+^CD8^−^ cells expressing IFNγ in PBMC treated with Tofacitinib (Fig. 2B, *p* < 0.05), and a trending decrease for granulocyte–macrophage colony stimulating factor (GM-CSF) (*p* = 0.06), of these, the decrease in GM-CSF displayed a trending correlation with DAS28-CRP (Supplementary Table 1). The decrease in IFNγ was also observed in CD4^+^CD8^+^ (Fig. 2C, *p* < 0.05), and at even a greater effect in CD4^−^CD8^+^ T cells (Fig. 2D,* p* < 0.0001), with a decrease also observed in Treg cells (Supplementary Fig. 4D). In CD4^−^CD8^+^ T cells, a significant decrease in GM-CSF expression was also observed (Fig. 2D, * p* < 0.01). Interestingly, no decrease was observed in cells treated with Humira, thus suggesting that these effects are JAK/STAT-dependent (Supplementary Fig. 3C).

To evaluate the possible effect of Tofacitinib on T cell polyfunctionality in the periphery, we performed SPICE analysis. This demonstrated an overall decrease in polyfunctionality in cells treated with Tofacitinib (Fig. 2E–G and legends in Supplementary Fig. 5B). In CD4^+^CD8^−^ specific co-expression combinations, including GM-CSF^+^IFNγ^+^IL-17^+^TNFα^+^, GM-CSF^+^IL-17^+^TNFα^+^, GM-CSF^+^TNFα^+^ (*p* < 0.05) and IL-17^+^TNFα^+^ were impaired by Tofacitinib treatment (Fig. 2E and H, and Supplementary Fig. 5B). In CD4^+^CD8^+^ and CD4^−^CD8^+^ T cells, we observed a trending increase in the fraction of cells not producing cytokines (negative fraction), and a decrease in the GM-CSF^+^IL-17^+^TNFα^+^ cells (Fig. 2F and I, and Fig. 2G and J).

Overall, these data highlight a role for Tofacitinib in inhibiting pathogenic cytokine production and polyfunctionality of T cells, with specific differences across the subsets.

### Tofacitinib inhibits T cell activation in the synovium of patients with IA

**Fig. 3:**
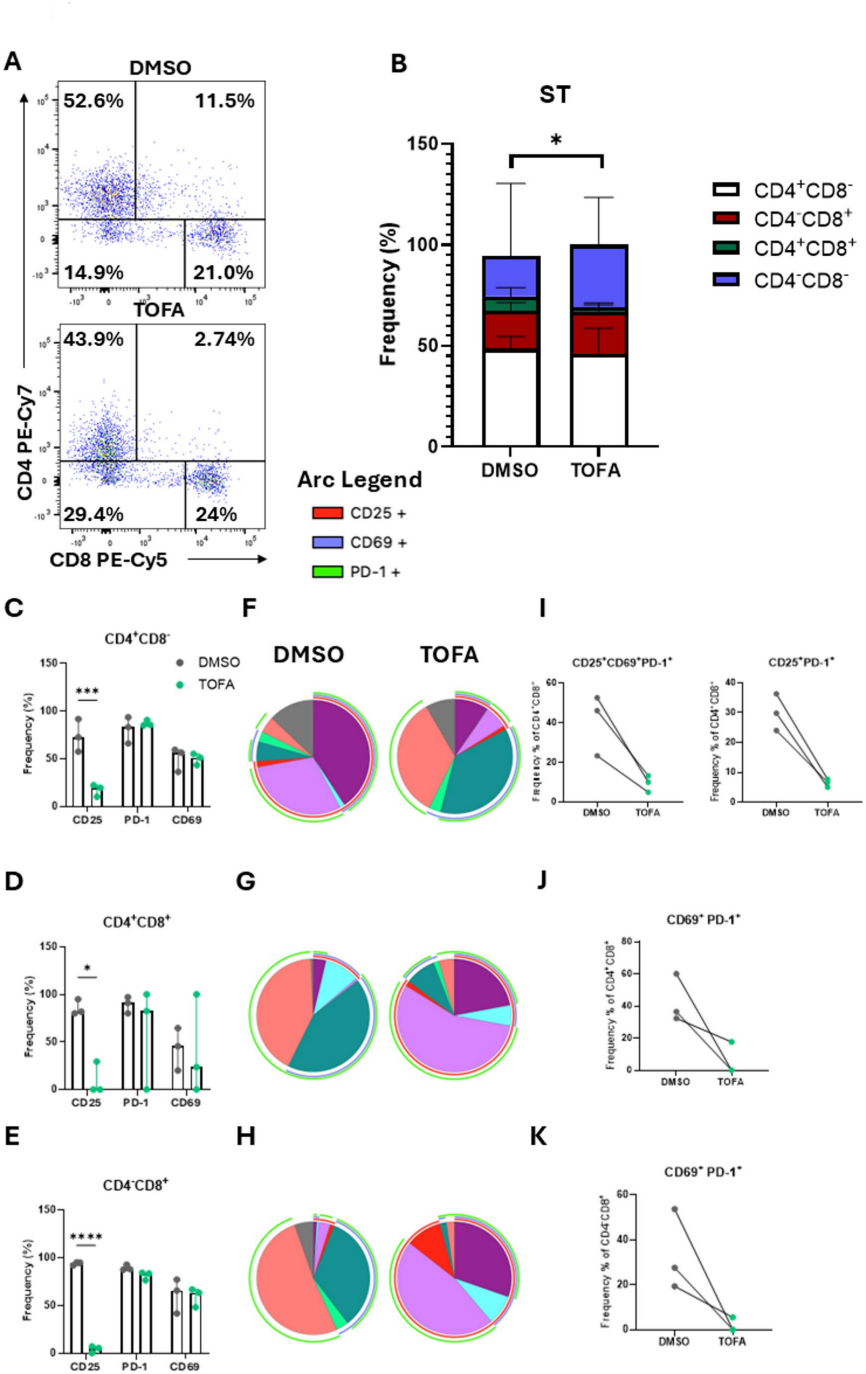
Tofacitinib inhibits synovial T cell activation in patients with IA. ST cell suspension was isolated from IA patients (n = 3; RA = 1, uIA n = 2) and treated with 5 µM Tofacitinib (or DMSO as control) in the presence of anti-CD3/anti-CD28 for 3 days. **A** Representative T cell population frequency dot plot and **B** Stacked bar chart (median with IQR) for CD4^+^CD8^−^, CD4^+^CD8^+^, CD4^−^CD8^+^ and CD4^−^CD8^−^ population frequencies (corresponding graphs in Supplementary Fig. 2B). Frequency of CD25, CD69 and PD-1 bar graphs (median with IQR) in **C** CD4^+^CD8^−^, **D** CD4^+^CD8^+^ and **E** CD4^−^CD8^+^ T cells (representative flow plot in Supplementary Fig. 2C). SPICE analysis for CD25, CD69 and PD-1 in **F** CD4^+^CD8^−^, **G** CD4^+^CD8^+^, and **H** CD4^−^CD8^+^ populations; overlapping arcs represent co-expression of markers (legend for pie chart in Supplementary Fig. 5A). Co-expression of different combinations of activation markers is expressed as median with IQR) in **I** CD4^+^CD8^−^, **J** CD4^+^CD8^+^, and **K** CD4^−^CD8^+^ populations. Differences among groups were evaluated by One-way or Two-way ANOVA with multiple comparisons (Bonferroni correction). For two group comparisons, paired Wilcoxon test was used. *p < 0.05. **p < 0.01, ***p < 0.001, ****p < 0.0001

Having established a role for Tofacitinib in decreasing T cell pathogenicity in the circulation, we sought to investigate its effects at the site of inflammation—the joints of patients with IA. ST suspensions were obtained as detailed in the methods and treated with 5 µM Tofacitinib (or DMSO as control) in the presence of anti-CD3/anti-CD28 for 3 days. After observing a shift in the frequencies of T cell subsets in the circulation, we demonstrate that this effect was maintained in the ST, with a significant decrease in the CD4^+^CD8^+^ population (Figs. 3A and B, and Supplementary Fig. 2B *p* < 0.01).

In addition, Tofacitinib significantly decreased the frequency of CD25-expressing CD4^+^CD8^−^ T cells (Fig. 3C,* p* < 0.001) and CD4^+^CD8^+^ (Fig. 3D,* p* < 0.05), and strongly in CD4^−^CD8^+^ (Fig. 3E,* p* < 0.0001). However, no significant effects were observed for CD69 and PD-1 expression across all three subsets. Interestingly, the decrease in CD25^+^ T cells did not result in a decrease of the Treg population, as observed in Supplementary Fig. 4C. When co-expression was evaluated by SPICE analysis (Fig. 3F–H and legends in Supplementary Fig. 5A), we observed a decrease in CD25^+^CD69^+^PD-1^+^ and in CD25^+^PD-1^+^ co-expression by the CD4^+^CD8^−^ T cell population (Fig. 3F and I). In addition, frequency of CD69^+^PD-1^+^ was decreased in CD4^+^CD8^+^ T cells (Fig. 3G and J) and CD4^−^CD8^+^ T cells (Fig. 3H and K), thus demonstrating a decrease in synovial T cell activation by JAK/STAT inhibition.

### Tofacitinib inhibits polyfunctionality of ST-derived T cells from patients with IA

**Fig. 4:**
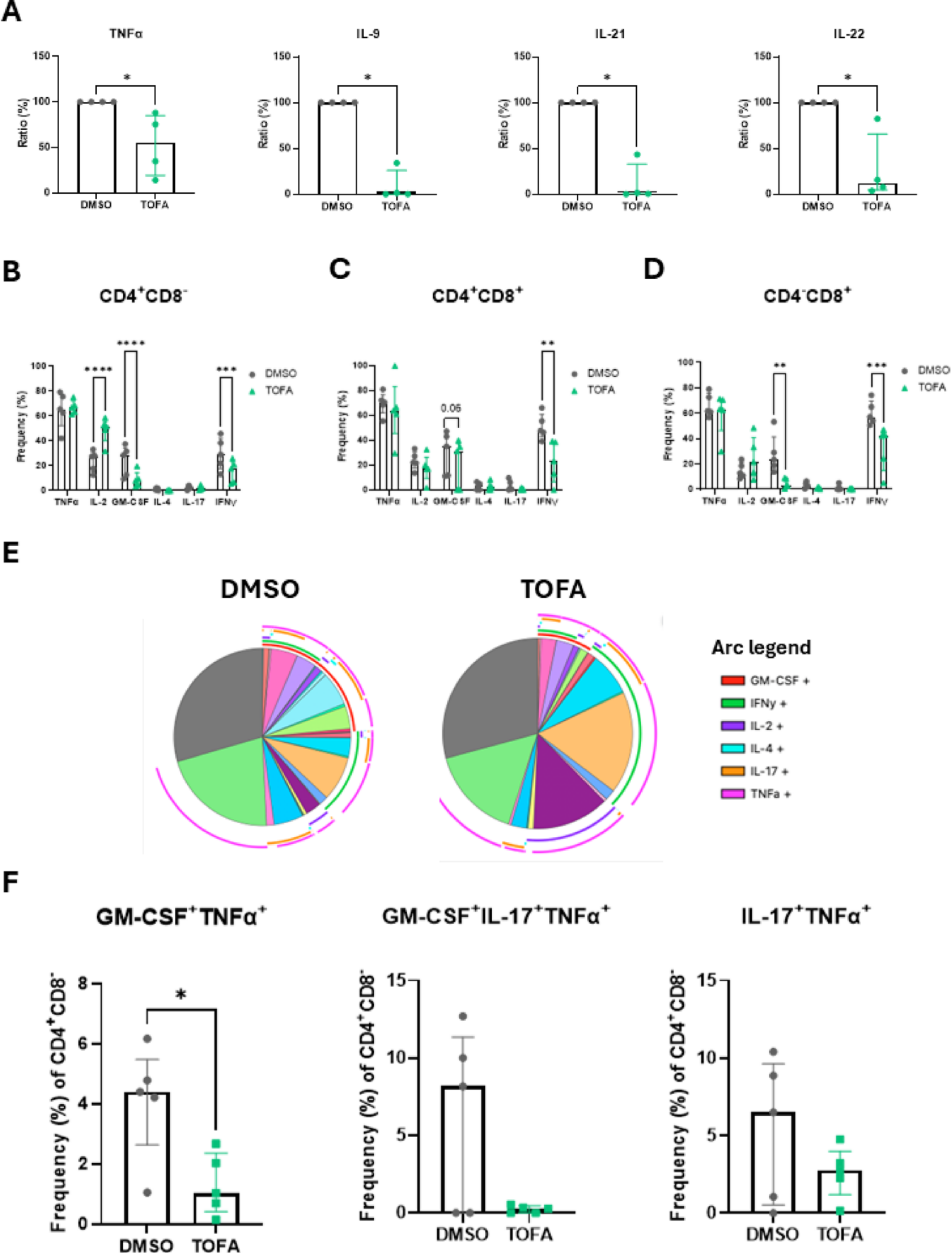
Tofacitinib decreases synovial T cell polyfunctionality in IA patients. ST cell suspension was isolated from IA patients (n = 5; RA n = 3, uIA n=2) and treated with 5 µM Tofacitinib (or DMSO as control) in the presence of anti-CD3/anti-CD28 for 3 days. **A** Supernatants were collected, and multiplex analysis was performed for TNFα, IL-21, IL-22, and IL-9. Data are normalised for DMSO (100%). **B**–**D** Cytokine release was stimulated with PMA/Ionomycin for 1 h, followed by BFA/Monensin for further 4 h. Surface and intracellular staining were performed for GM-CSF, IFN-γ, IL-2, IL-4, IL-17A, and TNFα. Bar charts are represented as median with IQR for **B** for CD4^+^CD8^−^, **C** CD4^+^CD8^+^, and **D** CD4^−^CD8^+^ T cells. SPICE analysis in **E** CD4^+^CD8^−^ are shown, with overlapping arcs representing co-expression of markers (legend for pie chart in Supplementary Fig. 5B). Co-expression of different combinations of activation markers is expressed as median with IQR in **F** CD4^+^CD8^−^ population. Differences among groups were evaluated by One-way or Two-way ANOVA with multiple comparisons (Bonferroni correction). For two group comparisons, paired Wilcoxon test was used. *p < 0.05. **p < 0.01, ***p < 0.001, ****p < 0.0001

We next evaluated soluble proteins in the supernatants of T cells isolated from ST by multiplex assay, stimulated as described above. Interestingly, Tofacitinib led to a significant decrease of multiple soluble cytokines, including TNFα, IL-9, IL-21 and IL-22 (Fig. 4A, all *p* < 0.05).

We next sought to investigate the effect of Tofacitinib on ST-derived T cell polyfunctionality by examining intracellular cytokines. A strong decrease in GM-CSF was observed in CD4^+^CD8^−^ (Fig. 4B,* p* < 0.0001), CD4^+^CD8^+^ (Fig. 4C,* p* = 0.06), and CD4^−^CD8^+^ T cell subsets (Fig. 4D,* p* < 0.01). In addition, IFNγ was also decreased in all three T cell populations: CD4^+^CD8^−^ (Fig. 4B,* p* < 0.001), CD4^+^CD8^+^ (Fig. 4C,* p* < 0.01), and CD4^−^CD8^+^ (Fig. 4D,* p* < 0.001). Minimal decrease in IFNγ was also observed in Tregs, although it did not reach significance (Supplementary Fig. 4F). In CD4^+^CD8^−^ T cells, an increase in IL-2-expressing cells (an early activation cytokine) was also observed (Fig. 4B,* p* < 0.0001). SPICE analysis demonstrated a decrease in ST-derived T cell polyfunctionality following Tofacitinib treatment as measured by specific cytokine combinations (Fig. 4E and F). Specifically, in CD4^+^CD8^−^ T cells, a notable decrease was observed for GM-CSF^+^TNFα^+^ (Fig. 4E and F,* p* < 0.05), GM-CSF^+^IL17^+^TNFα^+^ and IL17^+^TNFα^+^ co-expression. Together, these data demonstrate that Tofacitinib alters T cell polyfunctionality in the periphery and at the site of inflammation.

### Tofacitinib limits T cell functions

Having established that Tofacitinib treatment had a marked, direct effect on peripheral blood- and ST-derived T cell activation and polyfunctionality, we sought to investigate its effect on different key functions of T cells.

**Fig. 5:**
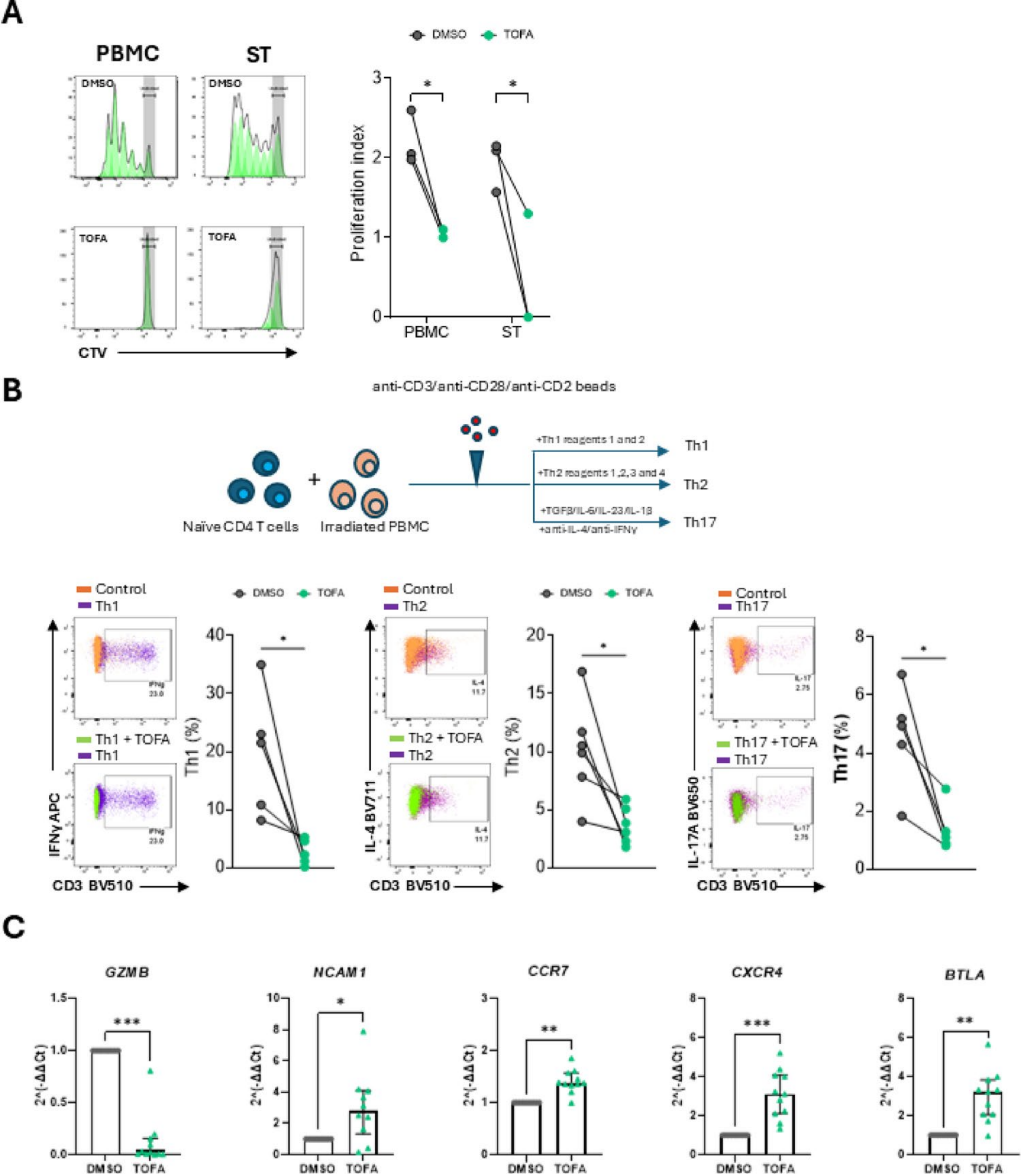
Tofacitinib alters T cell functions. **A** Flow cytometric analysis of IA patient PBMC and ST (n = 3; RA n = 2, PsA n = 1) treated with 5 µM Tofacitinib (or DMSO as control) and stimulated with anti-CD3/anti-CD28/anti-CD2 beads for 5 days. Representative gating strategy and proliferation tracking (left), and cumulative data of proliferation index (right). **B** Naïve CD4^+^ T cells were isolated from IA patient peripheral blood (n = 5; RA n = 4, PsA n = 1) and cultured in vitro with lethally irradiated heterologous PBMC under Th1, Th2 and Th17 polarising conditions. Representative flow cytometric analysis and cumulative data of Th1, Th2 and Th17 cell identification based on the expression of IFNγ (Th1), IL-4 (Th2) and IL-17A (Th17). Symbols indicate individual samples. **C** qPCR performed on pan T cells (n = 11; RA n = 4, PsA n = 3, uIA n = 4) treated with 5 µM Tofacitinib (or DMSO as control) for 24 h for *GZMB, NCAM1, CCR7, CXCR4* and *BTLA*. Differences among groups were evaluated by paired Wilcoxon test. *p < 0.05. **p < 0.01, ***p < 0.001, ****p < 0.0001

To examine the effect of Tofacitinib on T cell proliferation, PBMC and ST single-cell suspensions were labelled with a cell-tracking dye followed by stimulation with anti-CD3/anti-CD28/anti-CD2-coated beads. Interestingly, Tofacitinib inhibited proliferation of T cells in the peripheral blood and importantly, in the ST, as evidenced by the reduction in the proliferation index (Fig. 5A, *p* < 0.05).

We next examined the effect of Tofacitinib on naïve CD4^+^ T cell polarisation. Naïve CD4^+^ T cells were FACS-sorted from patients with IA and stimulated in vitro under Th1, Th2 and Th17 conditions (Fig. 5B). Interestingly, Tofacitinib inhibited T cell polarisation irrespective of polarising conditions, leading to significant reductions in Th1, Th2 and Th17 cell generation (Fig. 5B, all *p* < 0.05), thus suggesting a strong role in limiting T cell polarisation.

To investigate whether Tofacitinib also has a role in T cell cytotoxicity, magnetically sorted pan T cells were obtained and treated with Tofacitinib (or DMSO), as detailed in the methods. Interestingly, Tofacitinib treatment let to a significant decrease in Granzyme B (*GZMB*) expression (Fig. 5C, *p* < 0.001), which is in agreement with previous studies [20, 31].

Neural cell adhesion molecule (*NCAM1*) was significantly increased following Tofacitinib treatment (Fig. 5C, *p* < 0.05). Although classically considered a marker of natural killer (NK) cells, NCAM1 can also be expressed on certain T cell subsets such as “NKT-like cells” and γδ T cells [32–34]. The upregulation of *NCAM1* may indicate the expansion of or preferential survival of niche T cell populations with enhanced adhesion or tissue-migratory capacity. For NK cells, NCAM1 contributes to ICAM-1-dependent adhesion and trafficking [35], and it may serve a similar function in T cells under Tofacitinib-mediated conditions.

CCR7 and CXC chemokine receptor (CXCR)-4 are the two main chemokine receptors involved in T cell trafficking to the lymph nodes and sites of inflammation [36–38]. Previous studies have shown that in inflammatory diseases, CCR7^+^ T cells have a dual role: they are involved in the migration of T cells to the site of inflammation and in their retention within the tissue [39]. However, CCR7 expression has also been linked to T cell egression from inflamed tissue [40]. Interestingly, Tofacitinib treatment led to a significant increase in the expression of both *CCR7* (*p* < 0.01) and *CXCR4* (*p* < 0.001) (Fig. 5C), suggesting enhanced T cell egression from the tissue for antigen presentation, as also indicated by the increase in B- and T-lymphocyte attenuator (*BTLA*) expression following Tofacitinib treatment (Fig. 5C).

### Tofacitinib reinstates a quiescent metabolic state in T cells

Metabolic adaptation is a dynamic process with significant impact on all facets of T cell development and function. Early upon T cell receptor (TCR)-mediated activation, T cells change in size and morphology, and meet their new metabolic needs by transitioning from OxPhos to glycolysis [41, 42].

**Fig. 6:**
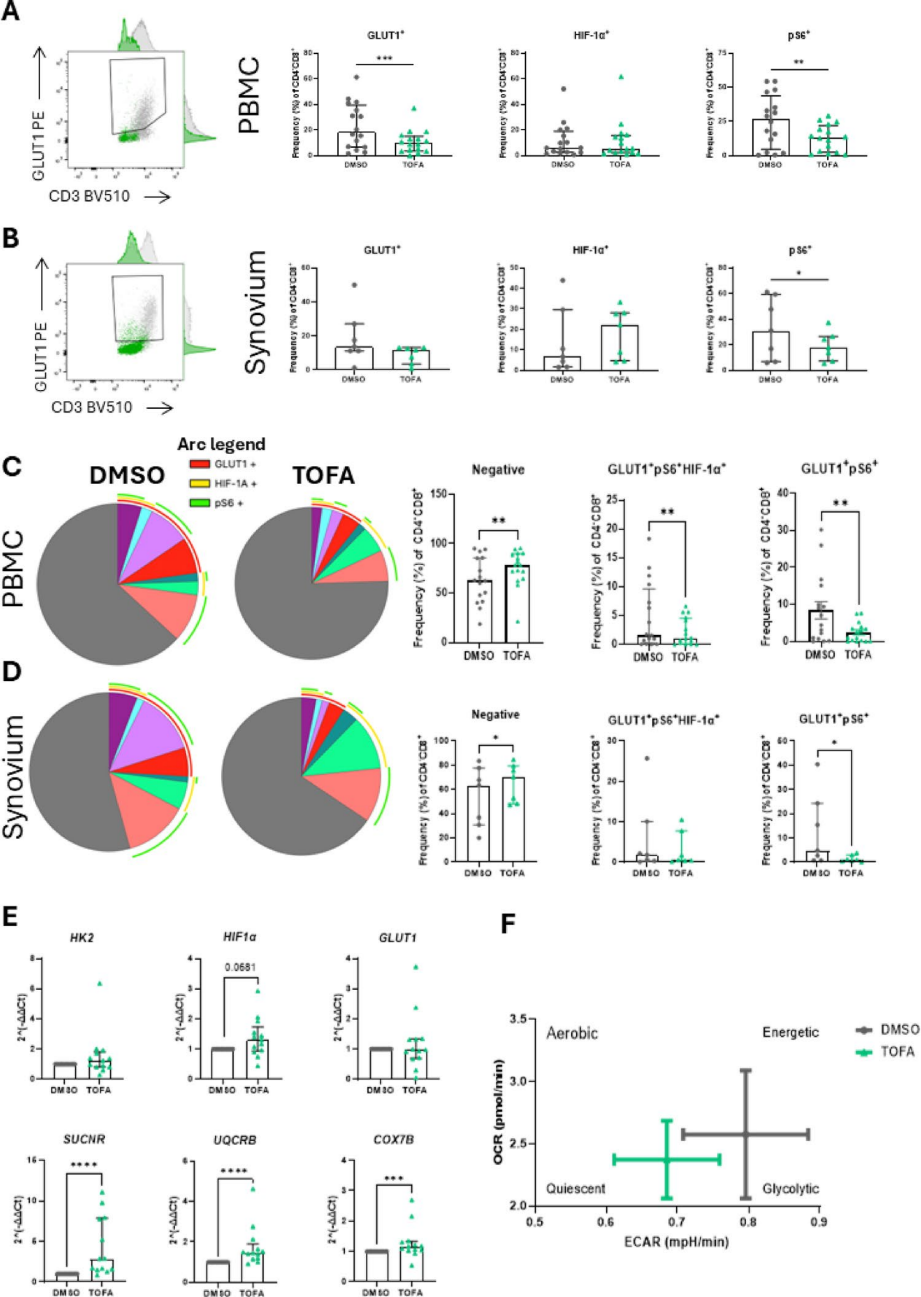
Tofacitinib reinstates a quiescent metabolic state in T cells. **A** PBMC (n = 16; RA n = 7, PsA n = 3, uIA n = 6) and **B** synovium (n = 7; RA n = 3, PsA n = 2, uIA n = 2) (ST n = 2, and SFMC n = 5) cell suspensions were isolated from IA patients and treated with 5 µM Tofacitinib (or DMSO as control) in the presence of anti-CD3/anti-CD28 for 3 days. Flow cytometric analysis was performed for metabolic markers GLUT1, HIF-1α and pS6 in the CD4^−^CD8^+^ T cell subset. SPICE analysis was performed on **C** PBMC and **D** synovium. Overlapping arcs represent co-expression of markers (legend for pie chart in Supplementary Fig. 6E). Co-expression of different combinations of activation markers is expressed as median with IQR. **E** qPCR performed on circulatory pan T cells (n = 13; RA n = 4, PsA n = 3, uIA n = 6) treated with 5 µM Tofacitinib (or DMSO as control) for 24 h for *HK2, HIF-1α, GLUT1, SUCNR1, UQCRB,* and *COX7B.* **F** Seahorse XF analysis was performed on pan T cells (n = 11; RA n = 5, PsA n = 1, uIA n = 5), and the energy map was calculated. Differences among groups were evaluated by paired Wilcoxon test. *p < 0.05. **p < 0.01, ***p < 0.001, ****p < 0.0001

To determine the metabolic profile of T cell subsets, flow cytometric analysis was performed on the glycolytic proteins glucose transporter 1 (GLUT1), HIF-1α and phosphorylated ribosomal protein S6 (pS6) [43, 44] in the three CD4/CD8 T cell populations. In the periphery, a significant decrease in GLUT1 (*p* < 0.001) and pS6 (*p* < 0.01) was observed in the CD4^−^CD8^+^ population in response to Tofacitinib treatment (Fig. 6A), while minimal effects were observed for the CD4^+^CD8^−^ population (Supplementary Fig. 6A). In the CD4^+^CD8^+^ population, a significant decrease in pS6 (*p* < 0.05) was observed (Supplementary Fig. 6B), with no effect on GLUT1 and HIF-1α. Thus, this suggests that the CD4^−^CD8^+^ population is the most sensitive to the metabolic switch induced by Tofacitinib. No effect was observed on Treg expression of metabolic markers in the periphery (Supplementary Fig. 4E). In addition, a decrease in GLUT1 (*p* < 0.001) and pS6 (*p* < 0.05) was also observed in circulating Th17 cells (Supplementary Fig. 6C). These inhibitory effects were also observed at the site of inflammation, the SF and the ST of patients with IA, albeit to a lower degree (Fig. 6B). Indeed, a decrease in pS6 was observed following Tofacitinib treatment (Fig. 6B, *p* < 0.05). Interestingly, a decrease in GLUT1 was also observed in synovium-derived Th17 cells (Supplementary Fig. 6D, *p* < 0.05). Both GLUT1 and HIF-1α were slightly decreased in the Treg population, although this did not reach significance (Supplementary Fig. 4G). SPICE analysis of the CD4^−^CD8^+^ cells revealed that the co-expression of markers was altered by Tofacitinib treatment (Fig. 6C and D, legends in Supplementary Fig. 6E). In the circulation, Tofacitinib increased the frequency of cells not expressing any markers (negative fraction in Fig. 6C, *p*  < 0.01), as well as decreasing the abundance of GLUT1^+^HIF-1α^+^pS6^+^ (*p* < 0.01) and GLUT1^+^pS6^+^-expressing cells (*p* < 0.01). In the synovium, we also observed an increase in the negative fraction (Fig. 6D, *p* < 0.05) and a decrease in GLUT1^+^pS6^+^ T cells (*p* < 0.05).

To confirm this metabolic switch was led by Tofacitinib treatment, we measured key genes involved in glycolysis and OxPhos in pan T cells. We observed that Tofacitinib induced a trending increase in gene expression associated with glycolysis, including hexokinase 2 (*HK2*), *HIF1α* (*p* = 0.068) and *GLUT1* (Fig. 6E). Interestingly, Tofacitinib also resulted in a strong increase in genes associated with OxPhos, including succinate receptor (*SUCNR*) (*p* < 0.0001), ubiquinol-cytochrome c reductase binding protein (*UQCRB*) (*p* < 0.0001) and cytochrome c oxidase (*COX7B*) (*p* < 0.001) (Fig. 6E). This suggests that Tofacitinib alters both metabolic pathways, with an enhanced effect on OxPhos. This was confirmed by Seahorse analysis, where both ECAR and OCR were quantified as measures of glycolysis and OxPhos, respectively. As compared to DMSO control, Tofacitinib induced a shift in the metabolic state of T cells towards a more quiescent state, as shown in the energy map in Fig. 6F.

Altogether, these data suggest that Tofacitinib induces a metabolic switch in T cells, which is more marked in circulation and mostly targeted at the CD4^−^CD8^+^ T cell subset.

## Discussion

Tofacitinib, a JAK/STAT pathway inhibitor, has been approved for the treatment of both RA and PsA, leading to the alleviation of pain and inflammation associated with IA [8, 45, 46]. Over the years, its efficacy has been studied, with a particular focus on its role in decreasing inflammation and pathogenicity of immune cells. Previous studies have demonstrated that Tofacitinib can inhibit the differentiation of monocyte-derived DCs in patients with RA and PsA and restrict T cell co-stimulatory capacity [17, 18, 47]. In addition, Tofacitinib has been shown to alter the Th17/Treg balance by inducing tolerogenic DCs in experimental autoimmune encephalomyelitis and limiting T cell proliferation [48]. In immune checkpoint inhibitor-induced arthritis, Tofacitinib has been shown to reduce synovial inflammation via the limitation of T cell activation [49]. However, little is known regarding the direct effect of Tofacitinib on T cell responses and polyfunctionality in patients with IA. Herein we evaluated the direct effect of Tofacitinib on T cell polyfunctionality, proliferation, polarisation, and metabolism.

Tofacitinib has been shown to have a direct effect in reducing T cell numbers in patients with RA [50, 51]. In agreement, here, we have demonstrated that Tofacitinib altered T cell distribution frequencies, with a specific reduction in the CD4^+^CD8^+^ population, which has been correlated with erosion and disease activity in RA [52]. Aberrant T cell activation and infiltration into the joint are key features of RA [29, 53, 54]. Studies have shown that activated T cells are enriched in the joints of patients with RA, with PD-1- and CD69-expressing cells correlating with disease progression [27, 28], while CD4^+^CD25^high^ T cell infiltration was independent of disease duration and erosion [29]. Here, we observe that JAK/STAT inhibition decreases T cell activation, both in the circulation and in the joints of patients with IA, leading to a reduction in CD25, PD-1 and CD69, alone or co-expressed, thus reducing the inflammatory profile of CD4^+^CD8^−^ and CD4^−^CD8^+^ subsets of T cells in IA disease. In addition, Tofacitinib limited T cell cytokine production, leading to a decrease of IFNγ and GM-CSF, but most importantly, their polyfunctionality, defined as the simultaneous expression of multiple pro-inflammatory cytokines. In fact, Tofacitinib decreased the co-expression of cytokines both in the circulation and in the joints of patients with IA, with an effect on all three CD4/CD8 T cell subsets. Interestingly in the tissue, Tofacitinib increased CD4^+^CD8^−^-derived IL-2, an early activator of T cells. This might be explained by the fact that while IL-2 production itself is governed by TCR-dependent transcriptional programs that are not directly controlled by JAK/STAT activity, inhibition of STAT5 by Tofacitinib may impair IL-2 consumption and feedback regulation, leading to intracellular accumulation of IL-2. Notably, increased IL-2 availability in this context may exert regulatory effects on T cell responses, including the maintenance of immune homeostasis [55, 56]. Importantly, ST-derived polyfunctional T cells have been shown to correlate with disease endotypes and progression in both RA and PsA [22, 23, 26], and are present in the tissue prior to the manifestation of clinical features of RA, therefore highlighting their role in disease pathogenesis. These effects were notably specific to JAK/STAT inhibition, as no effect was observed in the presence of the TNFα inhibitor Humira.

Together with T cell activation and polyfunctionality, we also assessed the effect of Tofacitinib on T cell proliferation and differentiation, demonstrating a reduction of these functions in patients with IA treated with the JAK/STAT inhibitor. In particular, we observed a reduction in T cell polarisation to Th1, Th2 and Th17 cells, and T cell proliferation, all of which are associated with IA pathogenesis [57–59]. Interestingly, Tofacitinib may increase migration into the lymph nodes or inflamed joints, as indicated by increased expression of *CCR7*, *CXCR4* and *NCAM1*. Tofacitinib also appears to limit their cytotoxic potential and exhaustion profile, as evidenced by a decrease in Granzyme B expression.

In an attempt to understand how Tofacitinib reduces T cell pathogenesis in IA, we studied its effect on T cell metabolism. It has been shown that upon TCR-mediated activation, T cells change in size and morphology, and meet their new metabolic needs by transitioning from OxPhos to glycolysis [41, 42]. Here, we observed that Tofacitinib led to an increase in genes associated with both glycolysis (*HK2*, *HIF-1α*, *GLUT1*), and OxPhos (*SUCNR, UQCRB*, *COX7B*). These data were also confirmed by Seahorse metabolic analysis, where T cells underwent metabolic reprogramming following JAK/STAT inhibition. When stratifying for the three main T cell subsets, a marked effect on metabolism was observed in CD4^−^CD8^+^ T cells, with a specific decrease in glycolysis-associated GLUT-1 and pS6 and in different combination of metabolic markers. This suggests that while Tofacitinib leads to an increase in both glycolysis and OxPhos in pan T cells, the specific stratification of the different T cell subsets highlights a clearer decrease in glycolysis observed for CD4^+^CD8^+^ T cells, suggesting that this T cell subset may be more susceptible to a metabolic switch following JAK/STAT inhibition. Although JAK/STAT inhibition has been shown to alter macrophage and FLS metabolism [9, 15, 60–62], to the best of our knowledge, this is the first time that a direct effect on T cell metabolism is shown. Future study should investigate dynamic metabolism of the specific T cell subsets by Seahorse analysis to confirm these data.

While we did not directly measure JAK/STAT signalling in T cells under our specific in vitro conditions, prior mechanistic work demonstrates that Tofacitinib directly inhibits cytokine-induced STAT phosphorylation in human T cells both in vitro and in vivo [63, 64]. This provides supporting evidence that Tofacitinib engages its canonical target in T cells under experimental conditions similar to ours, consistent with reduced downstream cytokine production.

Overall, this study demonstrated that JAK/STAT inhibition by Tofacitinib altered T cell polyfunctionality, proliferation, polarisation, and metabolism in patients with IA. Interestingly, the observed effects of Tofacitinib extended beyond the inflamed joints to the systemic circulation, indicating a broader immunomodulatory role. This systemic activity may contribute to the reduced pathogenicity of circulating T cells, thereby limiting their potential to exacerbate inflammation upon migration to the joint, ultimately attenuating joint pathology. This study advances our knowledge of the mechanism of action of Tofacitinib, suggesting it might be beneficial in patients exhibiting high levels of T cell polyfunctionality and synovial T cell involvement.

Limitations of the study: The effect of Tofacitinib on T cell distribution and cytokine production was not dependent on the specific patient treatment (Supplementary Fig. 7), however, the small cohort size within each experimental group limits the power to detect treatment-specific effects. Future studies with larger, stratified cohorts are therefore needed to fully assess the impact of previous therapies on this in vitro model. Another limitation is that the in vitro system used does not fully recapitulate the in vivo pharmacokinetics and pharmacodynamics of Tofacitinib. Nevertheless, previous clinical studies have demonstrated effective JAK/STAT inhibition in human T cells following tofacitinib treatment [59, 60], supporting the physiological relevance of our findings. Finally, the heterogeneity of the patient samples represents an additional limitation. The cohort included patients with undefined IA, RA, and PsA, with approximately 35% being seropositive. This heterogeneity may have contributed to variability in the observed responses. Future studies with larger, more homogeneous cohorts should investigate the influence of autoantibody status on the mechanism of action of Tofacitinib, as well as disease-specific effects.

## Supplementary Information

Below is the link to the electronic supplementary material.

Supplementary Material 1

## Data Availability

No datasets were generated or analysed during the current study.
