# Supplementary material for: JAK/STAT inhibition reprograms T cell activation and metabolism in inflammatory arthritis patients
Source: Inflamm Res. 2026 May 5;75(1):108. doi: 10.1007/s00011-026-02242-5 (PMC13144226; doi:10.1007/s00011-026-02242-5)
Supplement: Supplementary file 1 — Supplementary Material 1 [file 11_2026_2242_MOESM1_ESM.pptx]

## Slide 1
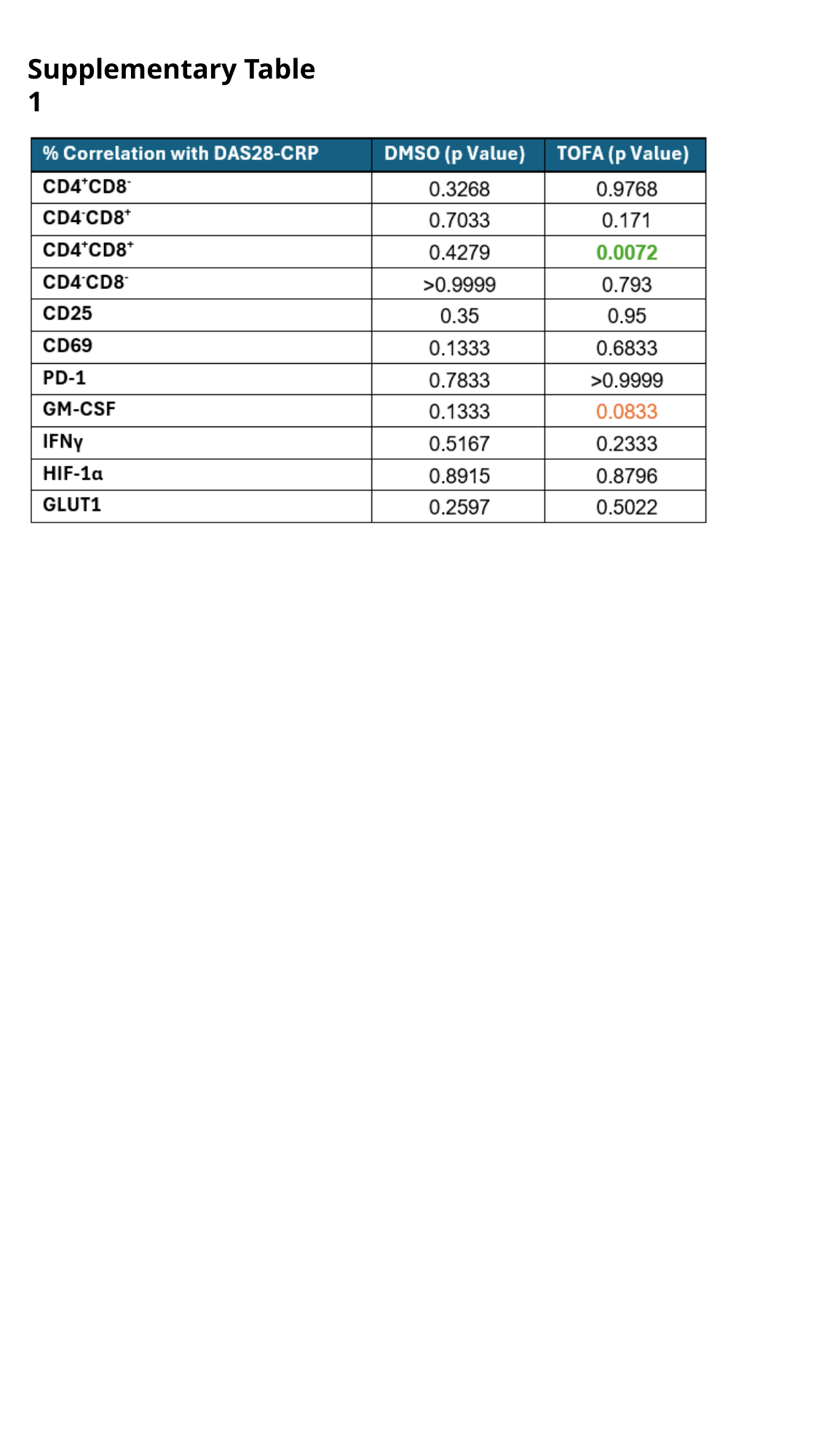

Supplementary Table 1

## Slide 2
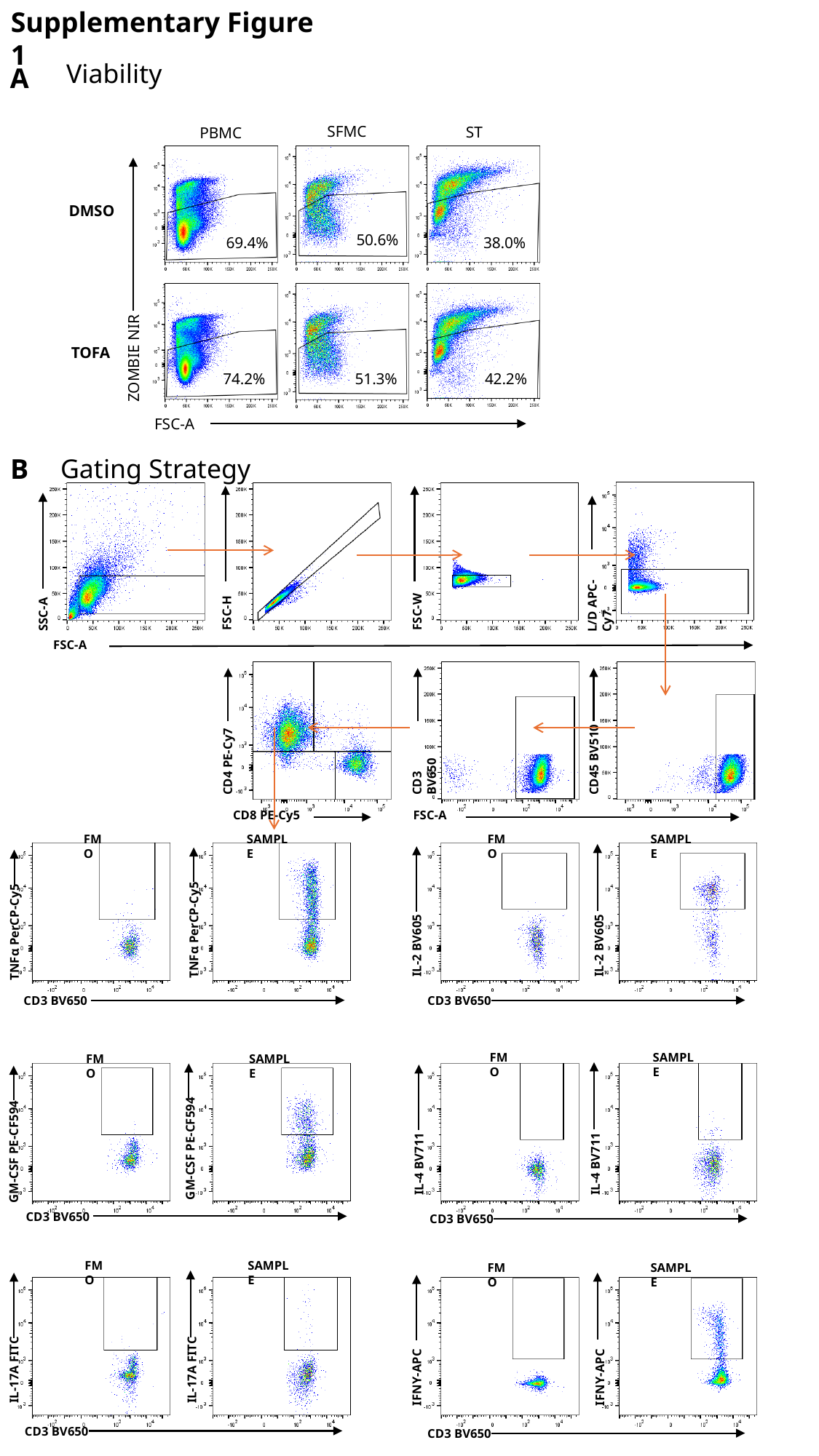

Supplementary Figure 1
Viability
A
SFMC
ST
PBMC
DMSO
50.6%
69.4%
38.0%
ZOMBIE NIR
TOFA
74.2%
51.3%
42.2%
FSC-A
B
Gating Strategy
L/D APC-Cy7
FSC-H
FSC-W
SSC-A
FSC-A
CD45 BV510
CD4 PE-Cy7
CD3 BV650
CD8 PE-Cy5
FSC-A
FMO
SAMPLE
FMO
SAMPLE
IL-2 BV605
IL-2 BV605
TNFα PerCP-Cy5
TNFα PerCP-Cy5
CD3 BV650
CD3 BV650
FMO
SAMPLE
FMO
SAMPLE
GM-CSF PE-CF594
GM-CSF PE-CF594
IL-4 BV711
IL-4 BV711
CD3 BV650
CD3 BV650
FMO
SAMPLE
FMO
SAMPLE
IL-17A FITC
IL-17A FITC
IFNΥ-APC
IFNΥ-APC
CD3 BV650
CD3 BV650

## Slide 3
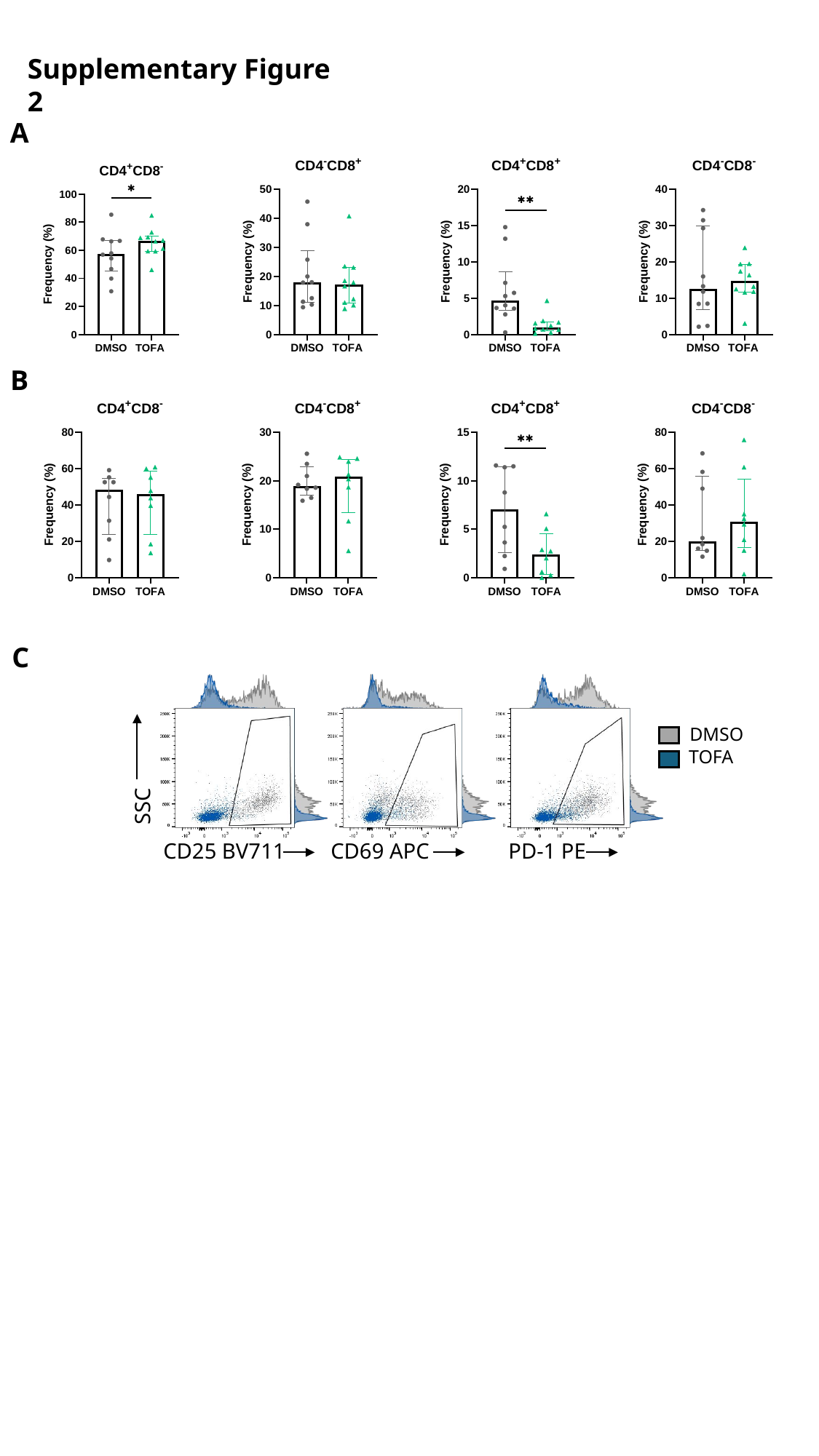

Supplementary Figure 2
A
B
C
DMSO
TOFA
SSC
CD25 BV711
CD69 APC
PD-1 PE

## Slide 4
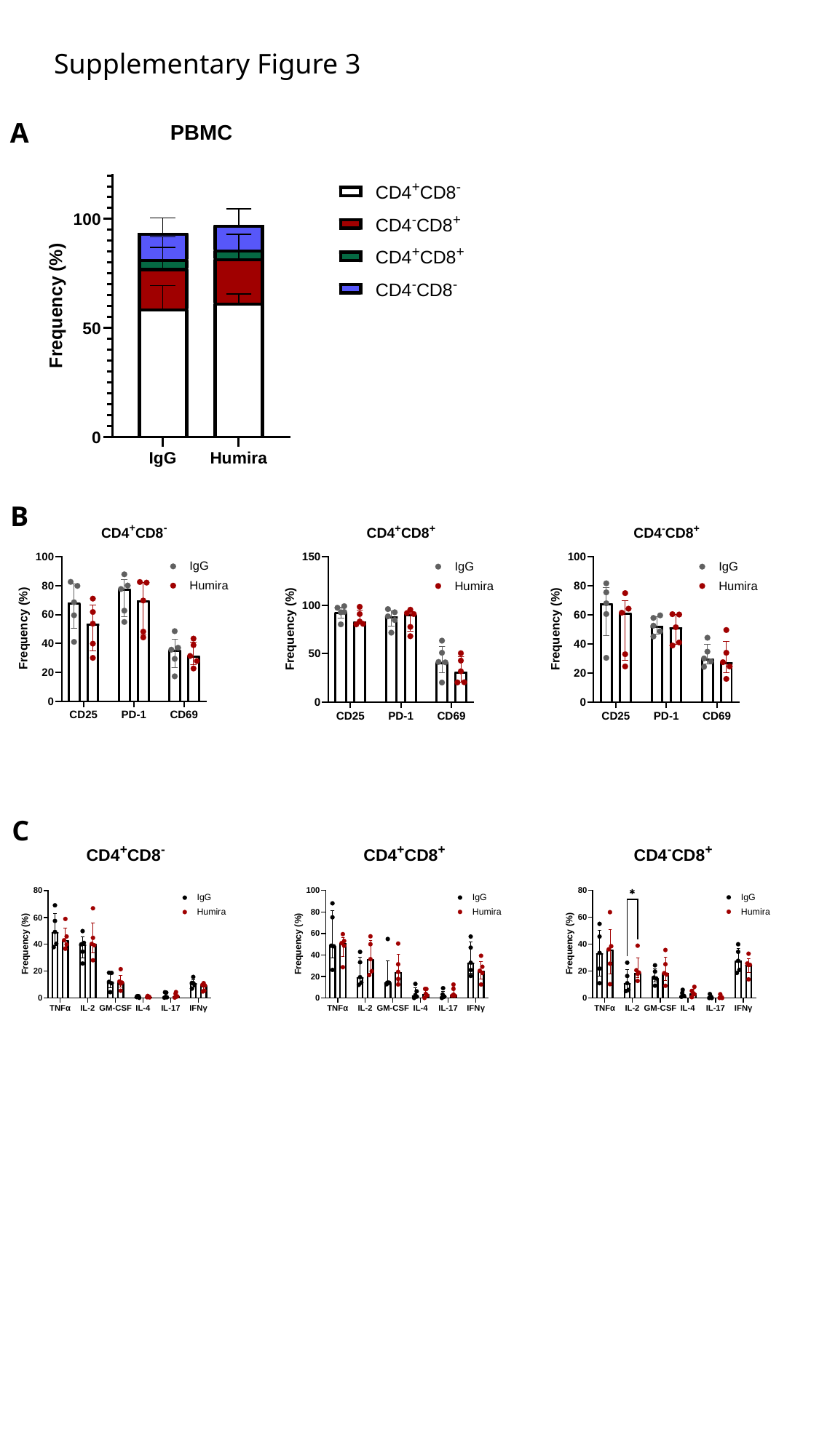

Supplementary Figure 3
A
B
C

## Slide 5
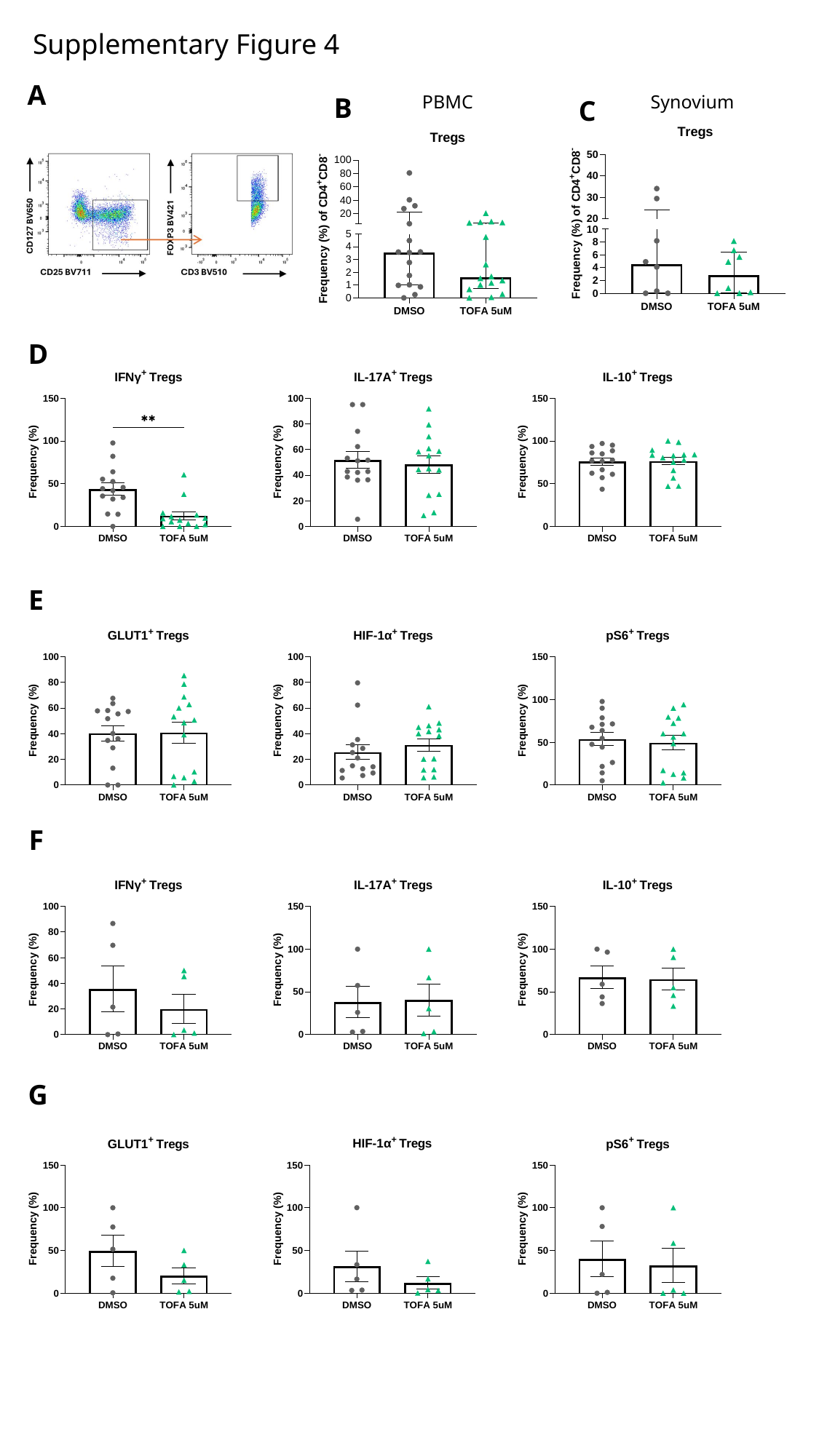

Supplementary Figure 4
A
B
PBMC
Synovium
C
D
E
F
G

## Slide 6
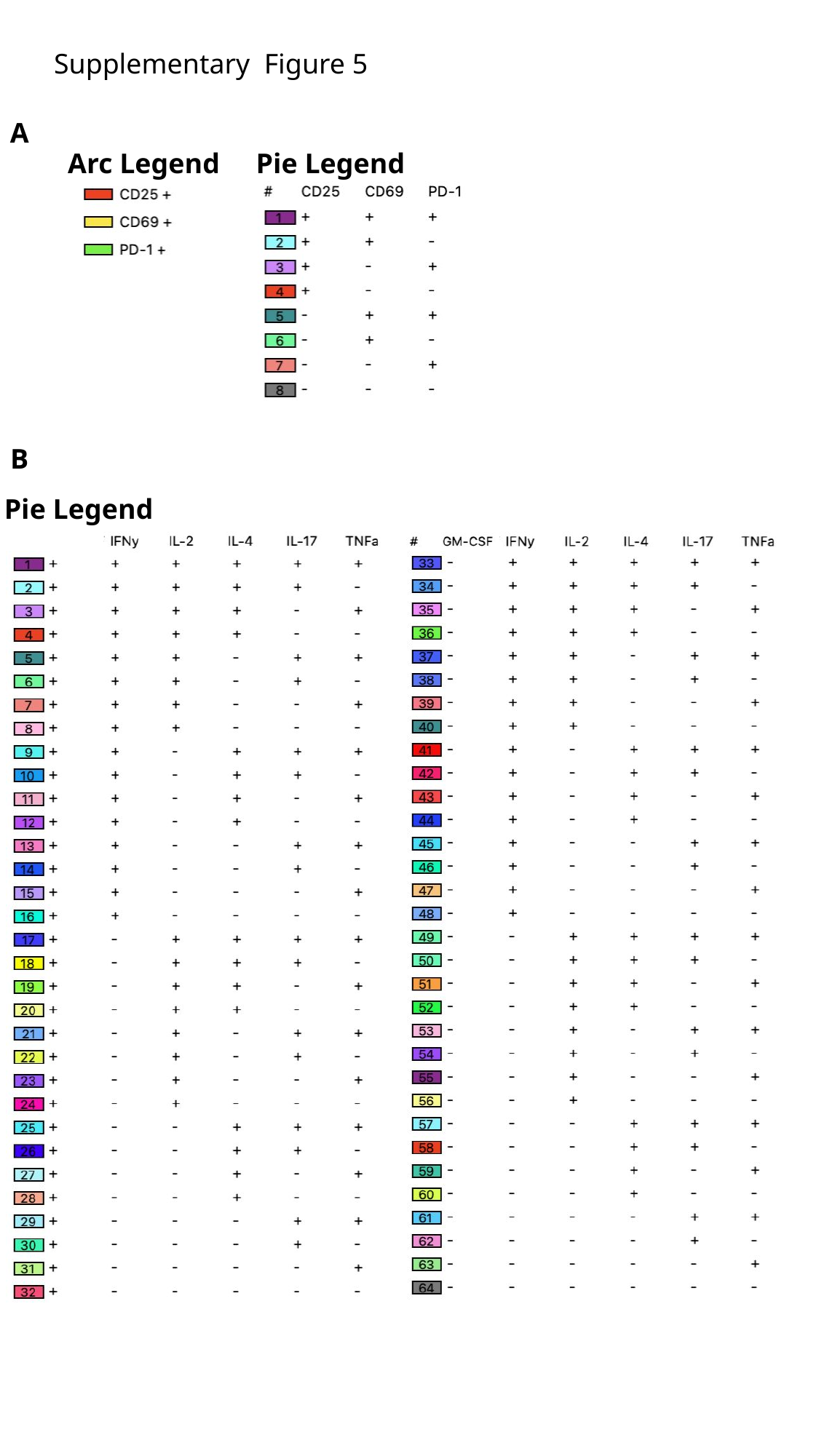

Supplementary Figure 5
A
Arc Legend
Pie Legend
B
Pie Legend

## Slide 7
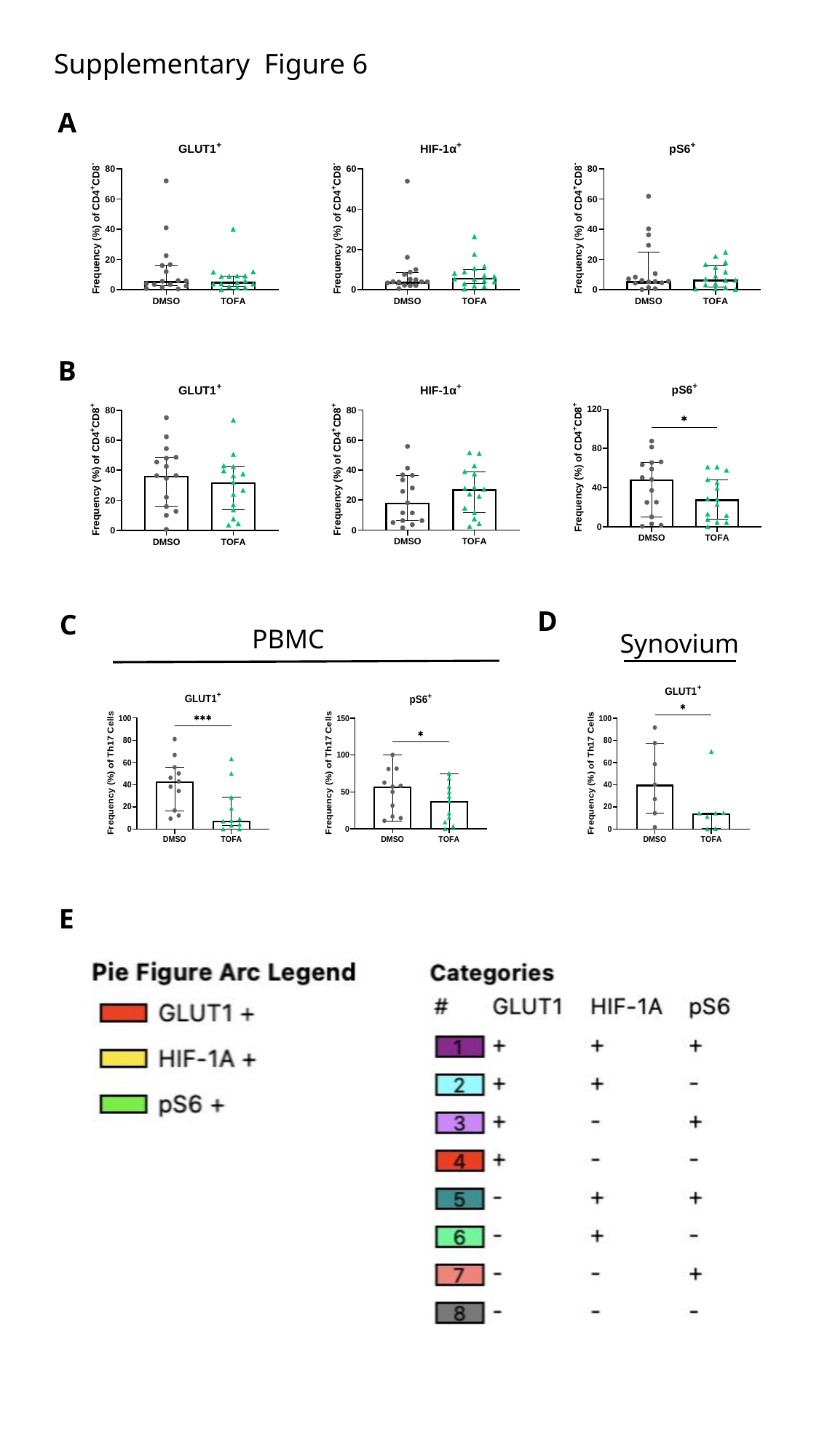

Supplementary Figure 6
A
B
D
C
PBMC
Synovium
E

## Slide 8
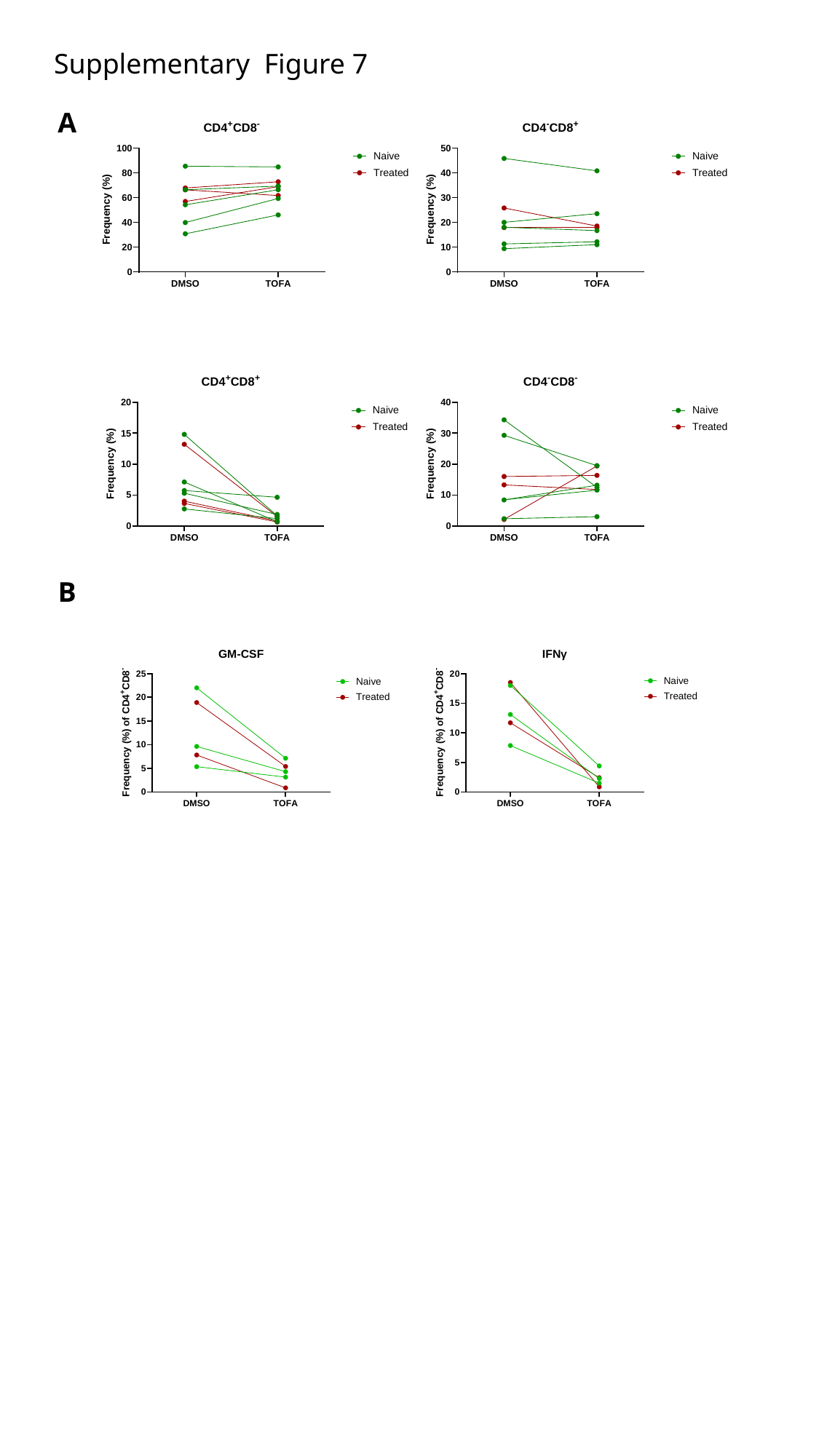

Supplementary Figure 7
A
B
